# HSP70-Hrd1 axis precludes the oncorepressor potential of N-terminal misfolded Blimp-1s in lymphoma cells

**DOI:** 10.1038/s41467-017-00476-w

**Published:** 2017-08-25

**Authors:** Wen-Fang Wang, Li Yan, Zhao Liu, Lan-Xuan Liu, Jian Lin, Zhi-Yin Liu, Xiong-Ping Chen, Wu Zhang, Zi-Zhen Xu, Ting Shi, Jun-Min Li, Yi-Lei Zhao, Guoyu Meng, Yi Xia, Jian-Yong Li, Jiang Zhu

**Affiliations:** 1 State Key Laboratory for Medical Genomics, Shanghai Institute of Hematology and Collaborative Innovation Center of Hematology, Rui-Jin Hospital Affiliated to Shanghai Jiao Tong University School of Medicine, Shanghai, 200025 People’s Republic of China; 2 State Key Laboratory of Microbial Metabolism, Joint International Research Laboratory of Metabolic and Developmental Science, School of Life Sciences and Biotechnology, Shanghai Jiao Tong University, Shanghai, 200240 People’s Republic of China; 3 School of Life Sciences and Biotechnology, Shanghai Jiao Tong University, Shanghai, 200240 People’s Republic of China; 4 Department of Laboratory Medicine, Rui-Jin Hospital Affiliated to School of Medicine, Shanghai Jiao Tong University, Shanghai, 200025 People’s Republic of China; 5 The First Affiliated Hospital of Nanjing Medical University, Jiangsu Province Hospital, Nanjing, 210029 People’s Republic of China

## Abstract

B lymphocyte-induced maturation protein-1 (Blimp-1) ensures B-cell differentiation into the plasma cell stage, and its instability constitutes a crucial oncogenic element in certain aggressive cases of activated B cell-like diffuse large B-cell lymphoma (ABC-DLBCL). However, the underlying degradation mechanisms and their possible therapeutic relevance remain unexplored. Here, we show that N-terminal misfolding mutations in ABC-DLBCL render Blimp-1 protein susceptible to proteasome-mediated degradation but spare its transcription-regulating activity. Mechanistically, whereas wild-type Blimp-1 metabolism is triggered in the nucleus through PML-mediated sumoylation, the degradation of lymphoma-associated mutants is accelerated by subversion of this pathway to Hrd1-mediated cytoplasmic sequestration and ubiquitination. Screening experiments identifies the heat shock protein 70 (HSP70) that selects Blimp-1 mutants for Hrd1 association, and HSP70 inhibition restores their nuclear accumulation and oncorepressor activities without disrupting normal B-cell maturation. Therefore, HSP70-Hrd1 axis represents a potential therapeutic target for restoring the oncorepressor activity of unstable lymphoma-associated Blimp-1 mutants.

## Abstract (web summary)

The transcriptional repressor Blimp-1 has an important role in B-cell differentiation. Here the authors show that lymphoma-associated Blimp-1 mutants are selectively recognized by HSP70-Hrd1, which leads to their accelerated degradation and propose HSP70 inhibition as a therapeutic approach for certain lymphomas.

## Introduction

B lymphocyte-induced maturation protein-1 (Blimp-1) or PR/SET domain 1 (PRDM1) is a master transcriptional repressor that governs the differentiation of embryonic stem cells into primordial germ cell-like cells and the development or maturation of numerous types of somatic tissues^1–5^. Recent studies have highlighted a critical role of Blimp-1 in the regulation of differentiation of innate and adaptive immune cells and the functional responses of these cells under pathological conditions^6–11^. Accumulating evidence indicates Blimp-1 insufficiency as being oncogenic in multiple types of lymphoid malignancy^12–19^. Specifically, the timely expression of physiological levels of Blimp-1 drives the terminal plasma cell differentiation of B cells through the preplasmablast stage primarily by transcriptionally extinguishing the expression of the gene sets characteristic of B lymphocytes^6, 20–23^. In contrast, the disruption of Blimp-1 expression and/or function arrests B-cell differentiation at the preplasmablast stage, and this effect, together with abnormal NF-κb activation, drives the initiation and progression of the activated B cell-like diffuse large B-cell lymphoma (ABC-DLBCL), a common subtype of aggressive lymphoma that is typically refractory to current therapies^12, 13, 24, 25^.

The extensive loss of Blimp-1 protein, as assayed by western blotting and immunochemical staining, has been documented in 63–77% of ABC-DLBCL cases^13, 19^. Initial studies have emphasized a variety of complex genetic and epigenetic abnormalities that disrupt the coding sequence or block the transcription of *Blimp-1* alleles^12, 13, 26, 27^. Nevertheless, in a fraction of ABC-DLBCL cases, a discordant elevation in the *Blimp-1* mRNA level is accompanied by a markedly decreased level of full-length Blimp-1 protein, thus indicating greatly increased Blimp-1 protein instability^1^. Specifically, the increased instability of four types of homogenously expressed Blimp-1 mutants carrying a single missense mutation (P84T, P84R, I107K, or Y185D) has been experimentally demonstrated^13, 28, 29^. The resulting Blimp-1 insufficiency is highlighted by the observation that Blimp-1 P84R and Y185D mutants, unlike WT Blimp-1, do not induce the plasma cell differentiation of BJAB cells (another subtype of DLBCL cells, namely the GCB-like DLBCL cells in which malignant B cells are differentiationally arrested at the germinal center stage) by suppressing the expression of Blimp-1 target genes. Critically, reintroduction of these mutants also cannot rescue the deficient plasma cell differentiation of *Blimp-1* ^−/−^ B cells in vivo^13^. However, the mechanism underlying this ABC-DLBCL-associated instability of Blimp-1 mutants has not been elucidated. Moreover, a large cohort study has recently revealed that the specific missense mutations clustered within the N-terminal coding region (exon 1-2) of *Blimp-1*, along with the greatly decreased expression of Blimp-1 protein, are correlated with poor prognosis in patients with ABC-DLBCL^19^. Nevertheless, how these specific N-terminal missense mutations underlie the loss of Blimp-1 protein has not been defined molecularly.

Hrd1, a multi-pass endoplasmic reticulum (ER) membrane protein, was originally identified as a ubiquitin (Ub) E3 ligase within an ER membrane-anchored complex that selects luminal misfolded glycoproteins for ER-associated degradation^30, 31^. In line with the more recent finding that its E3 ligase/RING domain is exposed to the cytosol, an additional role of Hrd1 in modifying certain cytoplasmic proteins, such as p53, and consequently inhibiting their nuclear entry and transcriptional regulatory activities has been shown^32^. Likewise, Hrd1 catalyzes the ubiquitination and proteasome-mediated degradation of normal Blimp-1 in dendritic cells (DCs) in response to Toll-like receptor signaling^33^, in agreement with the observation that Hrd1 accumulation is involved in the pathogenesis of rheumatoid arthritis^34^. Similarly, the SCF^DRE-1/FBXO11^ complex catalyzes the ubiquitination and degradation of Blimp-1 during the embryonic transition to adult in *C. elegans* ^35^. Nevertheless, because the Sumo-specific peptidase SENP1 greatly increases the stability of Blimp-1, a Sumo-1-dependent degradation pathway for normal Blimp-1 in the nucleus has been assumed to exist^36^. Adding to the controversy, a more recent study provided evidence that the attachment of Sumo-1 to Blimp-1, as catalyzed by the E3 ligase PIAS1, promotes the transcriptional regulatory activity of Blimp-1 without compromising its protein stability^37^. Therefore, the exact mechanisms mediating the metabolism of wild-type (WT) Blimp-1, and how they are involved in the degradation of lymphoma-associated Blimp-1 mutants, remain unclear.

In this study, our biochemical analyses of the metabolism of WT Blimp-1 and the unstable Blimp-1 mutants detected in ABC-DLBCL cell lines and primary ABC-DLBCL samples indicate that the instability of ABC-DLBCL-associated Blimp-1 mutants is largely due to their N-terminal misfolding mutations. Although WT Blimp-1 is predominantly metabolized in the nucleus through promyelocytic leukem ia protein (PML)-catalyzed Sumo-2/3 additions, the degradation of mutant Blimp-1 proteins is largely subverted to a cytoplasmic pathway by the HSP70–Hrd1 axis in a Sumo-2/3-independent but ubiquitination-dependent manner. Interestingly, an HSP70 inhibitor exerts potent anti-tumor effects on the ABC-DLBCL cells in vitro and in xenografting models by restoring the nuclear accumulation of the unstable Blimp-1 mutants, thus suggesting that it may be a promising therapeutic in the clinical management of the specific ABC-DLBCL cases that express N-terminal misfolded and unstable Blimp-1 mutants.

## Results

### N-terminal misfolding mutations render Blimp-1 unstable

**Fig. 1:**
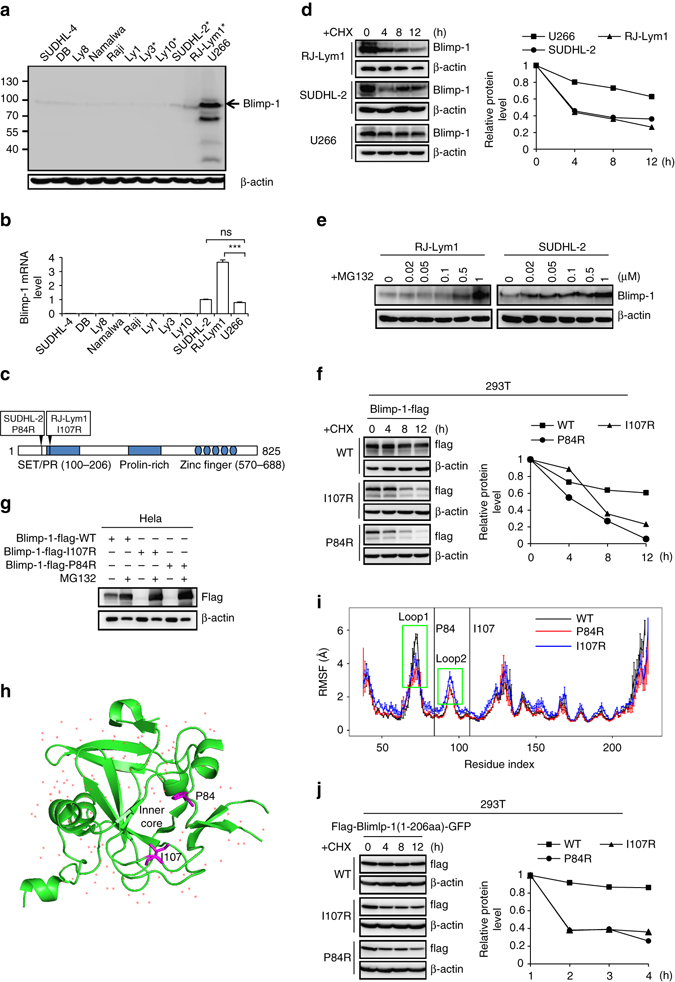
N-terminal misfolding mutations sensitize Blimp-1 to proteasome-mediated degradation in lymphoma cells. Blimp-1 protein (**a**) and mRNA (**b**) levels in B-cell lymphoma and MM cells were measured by western blotting and semi-quantitative RT-PCR, respectively. The ABC-DLBCL cells are indicated with *asterisks* (**a**). **c** Diagram of the Blimp-1 domain structure, with the location of P84R and I107R indicated. **d** The Blimp-1 protein half-life in RJ-Lym1, SUDHL-2, or U266 cells was measured by western blotting after treatment with 50 mg ml^−1^ CHX. The semi-quantitative protein levels are shown in the *right panel*. **e** The Blimp-1 protein level in RJ-Lym1 or SUDHL-2 cells was measured by western blotting 24 h after MG132 treatment at the indicated concentrations. **f** The half-life of flag-tagged WT Blimp-1, I107R mutant, or P84R mutant was measured as in **c** after 293T cells were transfected with the corresponding cDNAs. The semi-quantitative protein levels are shown in the *right panel*. **g** Western blot assay of the protein levels of WT or mutant Blimp-1 proteins expressed in HeLa cells exposed to MG132 treatment. **h** The slabbed view of the crystal structure of the WT N-terminal fragment (aa 38–223) with water locations shown. The P84 and I107 residues are shown in *magentas* and *stick* representation within the inner hydrophobic core. **i** The RMSF analyses of WT Blimp-1, the I107R mutant and the P84R mutant are shown in the *right panel* (values in Å). The positions of P84 and I107 are indicated with vertical lines. **j** The N-terminal fragments of WT or mutant Blimp-1 proteins (aa 1–206) were fused to GFP at its N terminus, and the half-lives of the fusion proteins were measured after expression in 293T cells as in **f**. Data are represented as mean ±SD. ***P < 0.01, *P < 0.05, *NS* no significance

We performed western blotting and RT-PCR assays on three ABC-DLBCL cell lines (Ly3, Ly10, and SUDHL-2)^13, 26^,Tam et al.^27^, one xenograft sample from a patient with ABC-DLBCL (identified as RJ-Lym1) (Supplementary Fig. 1a), one multiple myeloma (MM) cell line (U266), and six cell lines that represent other types of B-cell lymphoma to screen for unstable mutants of Blimp-1 proteins. As expected, abundant Blimp-1 protein was readily detected in only U266 cells (Fig. 1a), and its functionality was confirmed by expression analysis of several Blimp-1-regulated genes by using RT-PCR (Supplementary Fig. 1b). Nevertheless, the SUDHL-2 and RJ-Lym1 cells had *Blimp-1* mRNA levels comparable to or above that in the U266 cells (Fig. 1b), thus indicating that the Blimp-1 proteins in these ABC-DLBCL cells were potentially unstable. A previous study of SUDHL-2 cells has revealed a homogenously expressed *Blimp-1* mRNA transcript that encodes a particular Blimp-1 mutant (P84R)^13^. Analogously, sequencing analysis of the full-length *Blimp-1* mRNA transcript and its corresponding genomic exons in RJ-Lym1 cells also revealed a homogenously expressed mRNA transcript that harbored a single point mutation at nt 320; this transcript encoded another Blimp-1 mutant (I107R) (Fig. 1c and Supplementary Fig. 1c). Determination of the half-life of WT Blimp-1 in MM cells and two Blimp-1 mutants in ABC-DLBCL cells confirmed that both Blimp-1 mutants were unstable (Fig. 1d). Interestingly, the mutant Blimp-1 levels were restored by treatment with proteasome inhibitors such as MG132, but not lysosome inhibitors (Fig. 1e and Supplementary Fig. 1d). This finding indicates that the shortened half-lives of Blimp-1 mutants were caused by their increased susceptibility to proteasome-mediated degradation. Both Blimp-1 mutants showed similar shortened half-lives relative to that of WT Blimp-1 after ectopic expression in 293T cells, thus verifying that Blimp-1 instability was primarily attributable to these two missense mutations rather than to other potential ABC-DLBCL-associated abnormalities (Fig. 1f). As expected, the proteasome inhibitors, but not the lysosome or autophagy inhibitors, restored the levels of the Blimp-1 mutants to levels comparable to that of WT Blimp-1, when the proteins were expressed in HeLa or 293T cells (Fig. 1g and Supplementary Fig. 1e).

Notably, the I107R and P84R mutations are located within amino acids (aa) 1–206 of the Blimp-1 protein at its N terminus, which comprises an acidic region and a positively regulated (PR) domain; this fragment as a whole is relatively independent from the other regions of Blimp-1 in structure and function^1, 2, 38^. Interestingly, according to a solved three-dimensional (3D) structure of this fragment (MMDB ID: 66036/PDB ID: 3DAL), both mutations are located in the inner hydrophobic core (Fig. 1h). We performed molecular dynamic simulations and found that the root mean-square fluctuation (RMSF) value was significantly lower in loop 1 of both P84R and I107R, and was higher in loop 2 of I107R compared with those values in WT (*P* < 0.01) (Fig. 1i). Thus, both mutations were implicated as misfolding mutations in the aa 1–206 fragment. When fused to the N terminus of GFP, the mutated aa 1–206 fragment, but not the WT fragment, rendered the fusion proteins unstable (Fig. 1j), thus indicating a dominant, degron-like role of these two mutated fragments in the whole-fusion proteins. As evidence against the possibility that this destabilizing effect is strictly dependent on the R aa substitution, swapping the aa to K at P48/I107 or even G (I107G) also significantly increased the susceptibility of Blimp-1 to proteasomal degradation (Supplementary Fig. 1f, g).

### WT and mutant Blimp-1s are metabolized via two pathways

**Fig. 2:**
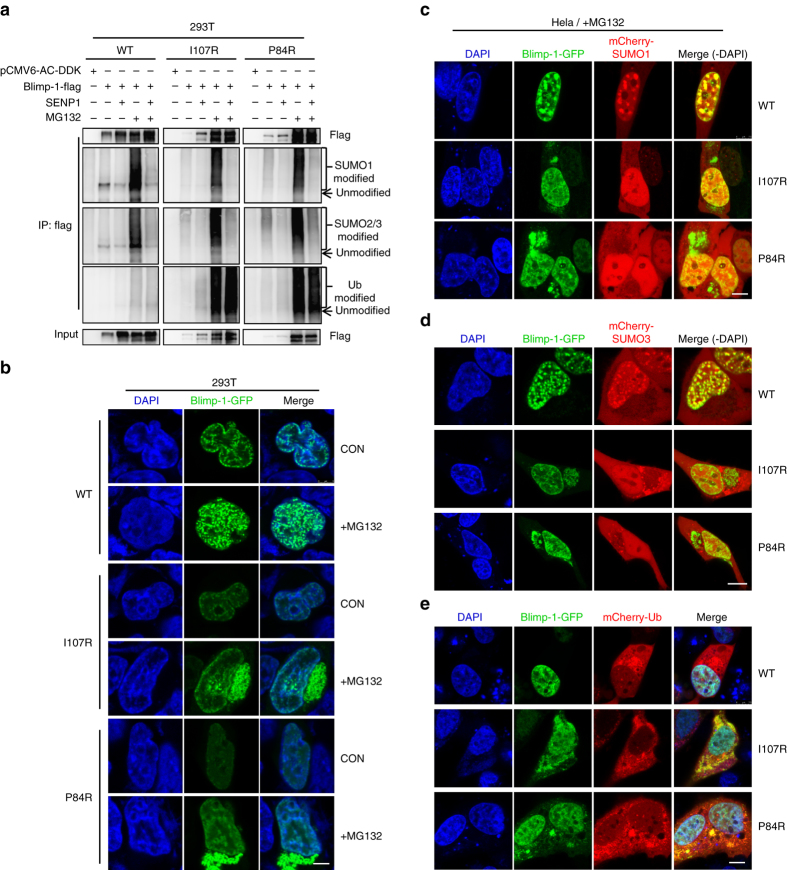
The primary modification and degradation sites differ for WT and mutant Blimp-1 proteins. **a** Flag-tagged WT or mutant Blimp-1-expressing plasmids were co-transfected with empty or SENP1-expressing plasmid into 293T cells, and then cells were treated with or without MG132 for 24 h. The lysates were immunoprecipitated (*IP*) with anti-flag antibody and then immunoblotted with antibodies against Sumo1, Sumo-2/3, and Ub. **b** 293T cells expressing GFP-tagged WT or mutant Blimp-1 proteins were treated with MG132 or left untreated for 12 h and observed under a confocal microscope. *Scale bar*: 5 μm. HeLa cells co-expressing GFP-tagged WT/mutant Blimp-1 proteins with mCherry-Sumo1 (**c**), mCherry-Sumo-3 (**d**), or mCherry-Ub (**e**) were treated with MG132 for 12 h and observed under a confocal microscope. *Scale bar*: 7.5 μm

We investigated how the degradation of Blimp-1 mutants differed from the metabolism of WT Blimp-1, which, as previously suggested^33, 36, 37^, potentially involves Sumo-1 or Ub modification. We initially observed that after MG132 treatment, the immunoprecipitated flag-tagged WT or mutant Blimp-1 proteins were modified not only by Sumo-1 and Ub^33, 36, 37^ but also by Sumo-2/3 (Fig. 2a). Concordantly, the immunoprecipitated myc-tagged Blimp-1 was directly modified by Sumo-2 in vitro (Supplementary Fig. 2a). Moreover, the Ub modification was much greater on the mutant Blimp-1 proteins than on WT Blimp-1 (Fig. 2a). Interestingly, the degradation of WT Blimp-1 largely depended on sumoylation, whereas the degradation of mutant Blimp-1 proteins only partially depended on this type of modification, because SENP1 overexpression, which removes Sumo-1 and Sumo-2/3 modifications, elevated WT Blimp-1 to a level comparable to that induced by MG132 but only slightly elevated the level of mutant Blimp-1 proteins (Fig. 2a). In contrast, SENP1 knockdown destabilized WT Blimp-1 (Supplementary Fig. 2b). In addition, Ub modification was relatively independent of Blimp-1 protein sumoylation, because Ub modification of WT and mutant Blimp-1 proteins was not decreased by SENP1 overexpression (Fig. 2a).

Microscopy and western blot assays showed that the WT and mutant Blimp-1 proteins were almost exclusively located within the nucleus in routinely cultivated HeLa and 293T cells (Fig. 2b and Supplementary Fig. 2c, d). Nevertheless, most of the noticeably accumulated mutant Blimp-1 proteins localized to the cytoplasm after MG132 treatment, whereas WT Blimp-1 remained within the nucleus at a moderately elevated level (Fig. 2b and Supplementary Fig. 2c, d). In parallel, the co-transfection of GFP-labeled Blimp-1 protein with mCherry-labeled Sumo-1, Sumo-3, or Ub showed that, in the absence of MG132 treatment, the nuclear WT or mutant Blimp-1 proteins colocalized only with Sumo-1 (Supplementary Fig. 2e–g). However, the Sumo-1 signals and the enhanced Sumo-3 signals colocalized with the accumulated nuclear, but not cytoplasmic, Blimp-1 proteins after MG132 treatment (Fig. 2c, d), whereas the Ub signals almost exclusively colocalized with cytoplasmic-mutant Blimp-1 proteins (Fig. 2e). Likewise, the Sumo-2/3 or Ub modification was detectable by western blotting in the P84R mutant after MG132 treatment (Supplementary Fig. 2h). Moreover, the Sumo-2/3-specific desumoylation enzyme SENP6 elevated Blimp-1 protein levels in a manner similar to SENP1 (Supplementary Fig. 2i). Altogether, these results indicated that the metabolism of WT Blimp-1 and a small fraction of each of the mutant Blimp-1 proteins is positively associated with Sumo-2/3 modification in the nucleus. However, a large fraction of mutant Blimp-1 enters into a sumoylation-independent, but ubiquitination-dependent, cytoplasmic pathway.

### PML-Sumo-2/3 axis controls Blimp-1 metabolism in nucleus

**Fig. 3:**
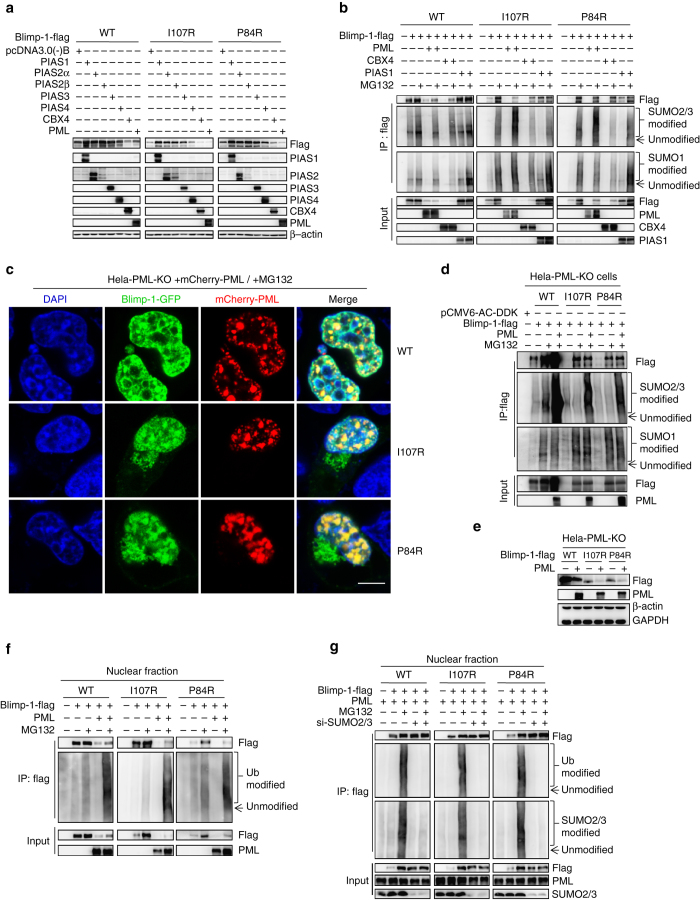
The metabolism of nuclear WT and mutant Blimp-1 proteins is triggered by PML-mediated Sumo-2/3 modification. **a** WT or mutant Blimp-1-expressing plasmids were co-transfected with empty plasmid (pcDNA3.0 (−) B) or PIAS1−, PIAS2α−, PIAS2β−, PIAS3−, PIAS4−, CBX4−, or PML-expressing plasmids into 293T cells. Total lysates were analyzed by western blotting. **b** WT or mutant Blimp-1-expressing plasmids were co-transfected with empty plasmid (pCMV6-AC-DDK) or PML−, CBX4−, or PIAS1−expressing plasmid into 293T cells, which were then treated with MG132 or left untreated for 12 h. The lysates were immunoprecipitated with anti-flag antibody and then immunoblotted with antibodies against Sumo-1 or Sumo-2/3. **c** *PML* ^−/−^ HeLa cells supplemented with mCherry-tagged PML were transduced with GFP-tagged WT or mutant Blimp-1-expressing plasmids and then treated with MG132 for 12 h before being inspected under a confocal microscope. *Scale bar*: 7.5 μm. **d** *PML* ^−/−^ HeLa cells with or without PML supplementation were treated with MG132 or left untreated for 24 h before whole-cell lysates were collected for immunoprecipitation and western blotting. **e** *PML* ^−/−^ HeLa cells with or without PML supplementation were transduced with flag-tagged WT or mutant Blimp-1-expressing plasmids for 48 h before being analyzed via western blotting. **f** WT or mutant Blimp-1-expressing plasmids were co-transfected with empty plasmid (pCMV6-AC-DDK) or PML-expressing plasmids into 293T cells and treated with MG132 or left untreated for 24 h. The nuclear lysates were immunoprecipitated with an anti-flag antibody and then immunoblotted with an antibody against Ub. **g** WT or mutant Blimp-1-expressing plasmids were transfected alone or together with si-SUMO-2/3 into PML-overexpressing 293T cells, which were then treated with MG132 or left untreated for 24 h. The nuclear lysates were immunoprecipitated with an anti-flag antibody and then immunoblotted with an antibody against Ub

Next, we explored the relevant mechanisms that mediate the Sumo-2/3 modification and degradation of nuclear WT and mutant Blimp-1 proteins. MG132 treatment clearly triggered the formation of insoluble aggregates when Blimp-1 accumulated (Supplementary Fig. 3a), and this phenomenon was predominantly observed with Blimp-1 mutants (Supplementary Fig. 3a)^39^. Nevertheless, co-immunoprecipitation (co-IP) of the soluble extracts detected the physical association of WT or mutant Blimp-1 proteins with multiple nuclear sumoylation E3 ligases, including PIAS1-4, PML and CBX4, but not RanBP2 (Supplementary Fig. 3a)^40^. Interestingly, PIAS1-4 overexpression elevated WT and mutant Blimp-1 protein levels, whereas PML or CBX4 overexpression decreased them (Fig. 3a). PIAS1 overexpression clearly did not increase the Sumo-2/3 modification of WT or mutant Blimp-1 proteins, regardless of the presence of MG132, and it is, in fact moderately increased the Sumo-1 modification of WT Blimp-1 when MG132 was added (Fig. 3b). However, PML (but not CBX4) overexpression increased the Sumo-2/3 modification of Blimp-1 proteins (particularly mutant proteins) without consistently increasing Sumo-1 modification (Fig. 3b).

The dot-like distribution pattern of the nuclear Blimp-1 proteins post MG132 treatment (Fig. 2b and Supplementary Fig. 2a) prompted us to examine whether these proteins actually accumulated within the PML nuclear bodies (PML-NB), subcellular organelles that have long been suspected of being involved in nuclear protein degradation^41, 42^. As expected, most of the nuclear WT or nuclear mutant Blimp-1 proteins were precisely localized within the PML-NB after MG132 treatment when they were fused with fluorescent proteins and ectopically expressed (Fig. 3c). Moreover, when PML was knocked out in HeLa cells, Sumo-2/3 (but not Sumo-1) modification was significantly decreased, whereas Blimp-1 protein levels were increased (Fig. 3d, e). However, MG132 treatment did not increase CBX4/Blimp-1 co-localization (Supplementary Fig. 3b), and CBX4 knockdown did not increase the Blimp-1 protein level (Supplementary Fig. 3c), thus excluding a major and direct role of CBX4 in mediating the turnover of nuclear Blimp-1 proteins. As expected, PIAS1 clearly colocalized with Blimp-1 proteins prior to MG132 treatment, and this colocalization was not enhanced by MG132 treatment (Fig. 2c and Supplementary Fig. 3d). Collectively, these results emphasized a fundamentally critical role of PML in triggering nuclear Blimp-1 metabolism by specifically mediating Sumo-2/3 modification.

In accordance with previous findings that RNF4 targets polymeric Sumo-2/3-modified nuclear proteins for ubiquitination and degradation^40^, a co-IP assay and microscopic observation showed that at least a fraction of RNF4 molecules physically associate with nuclear Blimp-1 proteins, especially after MG132 treatment (Supplementary Fig. 3a, e). Moreover, western blotting revealed that PML overexpression increased the Ub modification of nuclear WT and mutant Blimp-1 proteins (Fig. 3f), a result consistent with the microscopic evaluation showing that certain nuclear Ub signals indeed colocalized with WT or mutant Blimp-1 proteins and PML (Supplementary Fig. 3f). As expected, Sumo-2/3 knockdown dampened the PML-mediated Ub modification of nuclear Blimp-1 protein (Fig. 3g). These data indicated that at least a fraction of Sumo-2/3-modified nuclear WT or mutant Blimp-1 proteins is metabolized through a sumoylation-coupled ubiquitination pathway.

### Hrd1 sequesters mutant Blimp-1s for cytoplasmic degradation

**Fig. 4:**
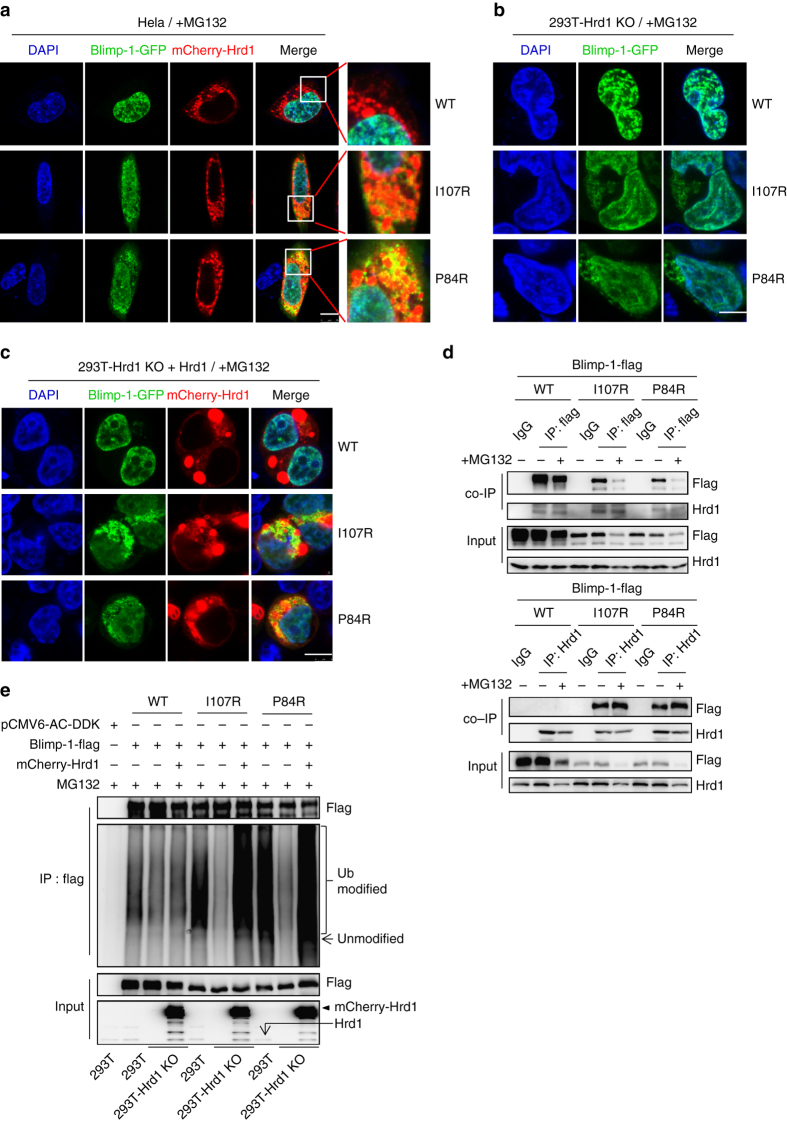
Endoplasmic Hrd1 selectively sequesters mutant Blimp-1 proteins, and promotes their ubiquitination and cytoplasmic degradation. **a** HeLa cells co-expressing mCherry-Hrd1 with GFP-tagged WT or mutant Blimp-1 proteins were treated with MG132 for 24 h and observed under a confocal microscope. *Scale bar*: 10 μm. **b** *Hrd1* ^−/−^ 293T cells expressing GFP-tagged WT or mutant Blimp-1 proteins were treated with MG132 for 12 h and observed under a confocal microscope. *Scale bar*: 7.5 μm. **c** *Hrd1* ^−/−^ 293T cells co-expressing mCherry-Hrd1 with GFP-tagged WT or mutant Blimp-1 proteins were treated with MG132 for 12 h and observed under a confocal microscope. *Scale bar*: 7.5 μm. **d** Flag-tagged WT or mutant Blimp-1 proteins were overexpressed in 293T cells, which were then treated with MG132 (20 μM) or left untreated for 12 h. The extracts were co-immunoprecipitated with control IgG or an anti-flag antibody (*upper panel*)/Hrd1 antibody (*bottom panel*). The co-immunoprecipitated lysates and extract input were then immunoblotted with antibodies against the indicated proteins. **e** Flag-tagged WT or mutant Blimp-1 proteins were overexpressed in 293T cells, *Hrd1* ^−/−^ 293T cells or *Hrd1* ^−/−^ 293T cells expressing mCherry-Hrd1. The cells were treated with MG132 for 24 h before extracts were immunoprecipitated with an anti-flag antibody and immunoblotted with an anti-Ub antibody

The results described above indicated that the degradation of mutant Blimp-1 proteins differs from that of WT Blimp-1 primarily in the increased susceptibility of the mutant proteins to cytoplasmic ubiquitination-mediated degradation. Immunofluorescence co-staining revealed that, after MG132 treatment, the accumulated mutant Blimp-1 proteins were distributed close to or partially merged with the outline of the ER (Supplementary Fig. 4a), and these proteins specifically localized with the outer membrane-anchored Hrd1 (Fig. 4a). Interestingly, most of the Blimp-1 mutants primarily translocated into the nucleus after MG132 treatment when *Hrd1* was deleted (Fig. 4b and Supplementary Fig. 4b), whereas the reintroduction of Hrd1 led to their redistribution back into the cytoplasm (Fig. 4c and Supplementary Fig. 4c). In contrast, the deletion or reintroduction of Hrd1 did not exert a detectable effect on the nuclear localization or level of WT Blimp-1 (Fig. 4b, c and Supplementary Fig. 4b, c). In accordance, a co-IP assay showed a much stronger association of Hrd1 with the mutant Blimp-1 proteins than with WT Blimp-1 (Fig. 4d). These results demonstrated a critical role of Hrd1 in selectively mediating the cytoplasmic sequestration of mutant Blimp-1 proteins. The crucial role of Hrd1 in mediating the ubiquitination of mutant Blimp-1 proteins was also evident from the result that Hrd1 deletion greatly decreased the Ub modification of mutant Blimp-1 proteins, but not WT Blimp-1, and that this reduction was reversed by exogenous Hrd1 expression (Fig. 4e).

### HSP70 selectively escorts mutant Blimp-1 proteins to Hrd1

Nevertheless, as previously suggested^33^, domain-mapping experiments suggested that Hrd1 recognizes aa 127–207 of the N-terminal fragment, which does not contain the P84R or I107R mutation (Supplementary Fig. 5a). The overlay of the representative structures of the WT and mutant Blimp-1 proteins (simulated) indicated that aa 127–207 do not include the mostly altered regions (loops 1 and 2 as shown in Fig. 1i) (Supplementary Fig. 5b). These results prompted us to examine whether additional factors that recognize the altered aa 1–130 subregion might contribute to the sequestration of mutant Blimp-1 proteins by Hrd1.

**Fig. 5:**
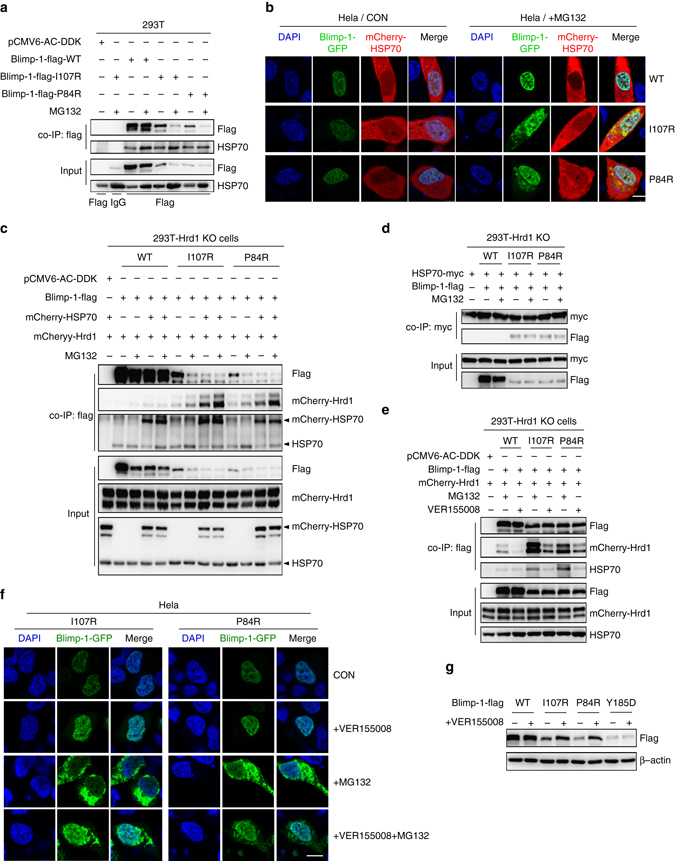
HSP70 selectively escorts mutant Blimp-1 proteins to Hrd1. **a** 293T cells overexpressing flag-tagged WT or mutant Blimp-1 proteins were treated with MG132 or left untreated for 12 h. The interaction between Blimp-1-flag and endogenous HSP70 was analyzed with a co-IP assay. **b** HeLa cells co-expressing mCherry-HSP70 with GFP-tagged WT or mutant Blimp-1 proteins were treated with MG132 or left untreated for 12 h and observed under a confocal microscope. *Scale bar*: 10 μm. **c** *Hrd1* ^−/−^ 293T cells co-expressing flag-tagged WT or mutant Blimp-1 proteins with mCherry-Hrd1 and mCherry-HSP70 were treated with MG132 or left untreated for 12 h. The lysates were co-immunoprecipitated with an anti-flag antibody and then immunoblotted with antibodies against the indicated proteins. **d** *Hrd1* ^−/−^ 293T cells co-expressing flag-tagged WT or mutant Blimp-1 proteins with myc-tagged HSP70 were treated with MG132 or left untreated for 12 h. The lysates were co-immunoprecipitated with an anti-myc antibody and then immunoblotted with antibodies against the indicated proteins. **e** *Hrd1* ^−/−^ 293T cells co-expressing mCherry-Hrd1 with flag-tagged WT or mutant Blimp-1 proteins were treated with MG132 or VER155008 for 12 h. Then, the cell extracts were co-immunoprecipitated with an antibody against flag and immunoblotted with the antibodies as indicated. **f** HeLa cells overexpressing GFP-tagged mutant Blimp-1 proteins were treated with MG132, VER155008 or both for 12 h and then observed under a confocal microscope. *Scale bar*: 10 μm. **g** 293T cells overexpressing flag-tagged WT or mutant Blimp-1 proteins were treated with VER155008 (10 μM) or left untreated for 24 h. Total lysates were analyzed by western blotting

We then performed mass spectrometric analysis of the co-IP lysates of WT and mutant Blimp-1 proteins with or without MG132 treatment. The results revealed that the heat shock protein HSP70 and several proteasomal subunits were significantly enriched in the co-IP lysate of the I107R Blimp-1 mutant compared with that of WT Blimp-1 during proteasome-mediated degradation (Supplementary Fig. 5c and Supplementary Table 1). In agreement with this result, a co-IP assay revealed that HSP70 exhibited a much stronger binding affinity to either mutant Blimp-1 protein than WT Blimp-1 (Fig. 5a), and microscopic analysis after MG132 treatment confirmed that HSP70 colocalized with the cytoplasmic Blimp-1 mutants but not with WT Blimp-1 (Fig. 5b).

Interestingly, although Hrd1, Blimp-1 mutants, and HSP70 were detected in the same complex (Supplementary Fig. 5d), a co-IP assay showed that the association of mutant Blimp-1 proteins with HSP70 was independent of the presence of Hrd1 (Supplementary Fig. 5e). Nevertheless, HSP70 overexpression promoted the physical association of Hrd1 with mutant Blimp-1 proteins, but not WT Blimp-1 (Fig. 5c, d), and in contrast, an HSP70 inhibitor disrupted the cytoplasmic sequestering effect of Hrd1 on mutant Blimp-1 proteins (Fig. 5e, f). These results indicated that prior association of Blimp-1 mutants with HSP70 may facilitate their recognition by Hrd1. Interestingly, the domain-mapping experiment showed that HSP70 exhibited a stronger binding affinity for mutant aa 1–130 than WT aa 1–130 or aa 127–207 (Supplementary Fig. 5f). In line with this, the aa 38–130 fragment, which was preferentially recognized by HSP70, showed a trend toward a more hydrophobic surface, whereas aa 127–207 did not (Supplementary Table 2). Moreover, the HSP70 inhibitor VER155008 selectively elevated the protein levels of P84R and I107R mutants but not the levels of WT or Y185D Blimp-1 (Fig. 5g). Altogether, these results suggested that the altered 3D structure of aa 38–130 containing the P84R or I107R mutations, but not the WT counterpart, is first picked up by HSP70, which in turn escorts Blimp-1 proteins to Hrd1, which specifically binds to the aa 127–207 subregion. In agreement with this possibility, an HSP70 inhibitor restored the nuclear accumulation of two other reported mutations occurring at P84 and I107, but not the mutation at Y185 (Supplementary Fig. 5g). Nevertheless, we did not find evidence that HSP70 escorts mutant Blimp-1 proteins to the C terminus of HSP70-interacting protein (CHIP), a well-documented HSP70 partner and an E3 ligase that mediates the ubiquitination and degradation of misfolded proteins (Supplementary Fig. 5h)^43^.

### HSP70 inhibition restores the function of mutant Blimp-1s

Previous studies have indicated that the N-terminal fragment (aa 1–206) of Blimp-1 is dispensable for its transcriptional regulatory activity^1, 2, 38^. Therefore, we tested whether the intrinsic transcriptional regulatory activity of Blimp-1 was retained in the P84R and I107R mutants. Interestingly, the overexpressed Blimp-1 mutants repressed the promoter activity of the target gene *CIITA* in a dose-dependent manner, and they functioned similarly to WT Blimp-1 when *Hrd1* was deleted (Supplementary Fig. 6a). In this regard, we then examined whether the transcriptional regulatory activity of other N-terminal (aa 1–130) missense mutation-associated unstable Blimp-1 mutants was restored by HSP70 inhibitors. As summarized in Supplementary Table 3, 6 out of 8 unstable Blimp-1 mutants were recognized by HSP70 and specifically degraded by the HSP70–Hrd1 axis (5 mutations occurring at the P84 or I107 sites). Accordingly, the nuclear transcriptional regulatory activities of P84 or I107 mutants were similarly restored by HSP70 inhibitors (Supplementary Fig. 6b). Overall, these observations identified the HSP70–Hrd1 axis as a relatively common pathway in mediating the degradation of N-terminally misfolded Blimp-1 mutants in primary ABC-DLBCL cases.

**Fig. 6:**
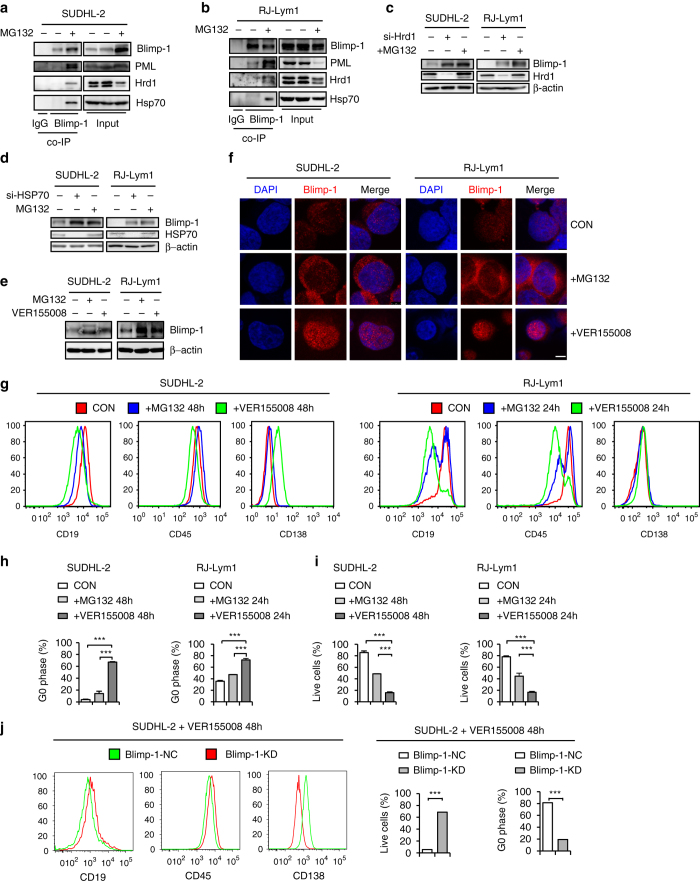
HSP70 inhibition increases the nuclear accumulation of mutant Blimp-1 proteins to suppress the proliferation of lymphoma cells. A co-IP assay was performed to evaluate the association of endogenous Blimp-1 mutants with PML, Hrd1 and HSP70 in SUDHL-2 (**a**) and RJ-Lym1 (**b**) lymphoma cells as indicated. SUDHL-2 or RJ-Lym1 cells were transfected with si-Hrd1 (**c**) or si-HSP70 (**d**) in parallel with those treated with MG132. Blimp-1 levels were measured by western blotting. **e** SUDHL-2 cells or RJ-Lym1 cells were treated with MG132 (1 μM) or VER155008 (30 μM) for 24 h, and Blimp-1 levels were measured by western blotting. **f** SUDHL-2 cells or RJ-Lym1 cells were treated with MG132 (1 μM) or VER155008 (30 μM) for 24 h, stained with a Blimp-1 antibody (*red*) and DAPI (*blue*), and then observed under a confocal microscope. *Scale bar*: 5 μm. **g** The plasma cell differentiation of SUDHL-2 cells or RJ-Lym1 cells was assessed by flow cytometry after treatment with MG132 or VER155008 for 48 or 24 h. The location in the cell cycle (**h**) or survival (**i**) of SUDHL-2 cells and RJ-Lym1 cells was assessed by flow cytometry after treatment with MG132 or VER155008 for 48 or 24 h. **j** SUDHL-2 cells stably transfected with control shRNA or with Blimp-1 shRNA were further treated with VER155008 for 48 h. The cell differentiation, survival, and location in the cell cycle were assessed by flow cytometry. Data are represented as mean ± SD. ***P < 0.01, *P < 0.05

A co-IP assay demonstrated that PML and Hrd1/HSP70 associated with mutant Blimp-1 proteins in lymphoma cells as they did in 293T and HeLa cells (Fig. 6a, b). Hrd1 or HSP70 knockdown elevated the level of the P84R and I107R mutants, as expected (Fig. 6c, d). Moreover, inducing the expression of the I107R mutant with Dox in Burkitt lymphoma Namalwa cells resulted in impaired cell proliferation and the repression of *Pax5*, *CIITA*, and *ID3* (Supplementary Fig. 6c–e). Therefore, we wondered whether restoring the stability of mutant Blimp-1 proteins might enable them to exert meaningful oncorepressor effects in ABC-DLBCL cells^21^. In this regard, proteasome inhibitors have been widely used in the treatment of ABC-DLBCL^44, 45^. Nevertheless, as predicted from the aforementioned results (Fig. 2b and Supplementary Fig. 2c), this treatment induced the cytoplasmic sequestration of the accumulated Blimp-1 mutants (Fig. 6f, *middle panel*), thus preventing them from exerting their transcriptional regulatory function. Moreover, MG132 increased XBP1 expression, as previously suggested (Supplementary Fig. 6f). Because XBP1 is known to upregulate Hrd1 expression^46, 47^, a vicious cycle may occur that blocks the nuclear translocation of the accumulated mutant Blimp-1 proteins.

Remarkably, when a similar total Blimp-1 level was restored in RJ-Lym1 or SUDHL-2 cells (Fig. 6e), VER155008, but not MG132, significantly restored the nuclear accumulation of Blimp-1 mutants (Fig. 6f). Accordingly, VER155008, but not MG132, decreased CD19 and CD45 expression and increased CD138 expression (Fig. 6g), an immunophenotypic change in accordance with possible plasma cell differentiation. This change was accompanied by the transcriptional repression of several Blimp-1-regulated genes (Supplementary Fig. 6g). In parallel, VER155008 exerted a much stronger inhibitory effect on the cell cycle and survival of these two lymphoma cell lines than MG132 (Fig. 6h, i). Importantly, Blimp-1 knockdown attenuated the anti-tumor effect of VER155008 in SUDHL-2 cells (Fig. 6j). Moreover, VER155008, but not MG132, also showed a stronger anti-proliferative effect on SUDHL-2 cells than Ly3 or Ly10 cells—two B-cell lymphoma lines with a disrupted Blimp-1 coding sequence (Supplementary Fig. 6h, i)^26^. Overall, these findings demonstrated that restoring the unstable mutant Blimp-1 proteins to the nucleus contributes to the anti-ABC-DLBCL effect independently of other possible effects of HSP70 inhibition.

### HSP70 inhibition suppresses growth of ABC-DLBCL xenografts

**Fig. 7:**
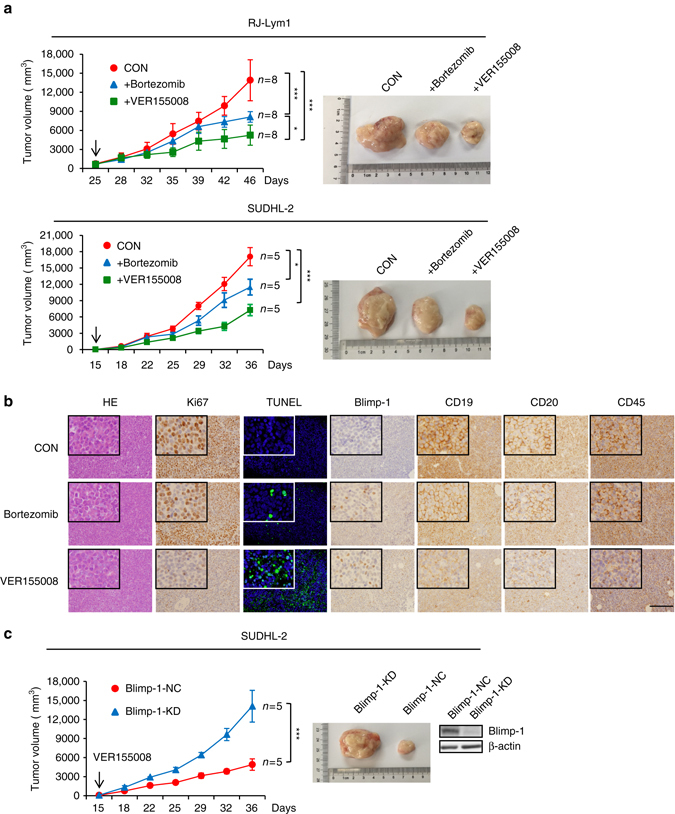
An HSP70 inhibitor suppresses the in vivo growth of ABC-DLBCL cells carrying unstable Blimp-1 mutants. **a** Mice in each cohort were treated with PBS (CON), bortezomib (1 mg kg^−1^) or VER155008 (40 mg kg^−1^) twice weekly for 3 weeks. Tumor volumes were measured every 3–4 days. Treatment began on the day indicated with an arrow. The representative gross morphology of the tumors isolated from the control group, the bortezomib treatment group, and the VER155008 treatment group are shown in the *right panel*. **b** The expression of Ki67, Blimp-1, CD19, CD20, and CD45 in the retrieved RJ-Lym1 tumor samples from the different groups was examined by IHC staining. HE staining and a TUNEL assay were performed in parallel. *Scale bar*: 50 μm. **c** SUDHL-2 cells stably transfected with control shRNA or with Blimp-1 shRNA were inoculated into NOD/SCID mice, which were treated with VER155008 (40 mg kg^−1^) as in **a**, and tumor volumes were measured every 3–4 days. The representative gross morphology of the tumors isolated from two groups and the western blot for Blimp-1 expression are shown in the *right panel*. Data are represented as mean ± SD. ***P < 0.01, *P < 0.05

Finally, we tested the therapeutic effect of VER155008 on the xenograft growth of human RJ-Lym1, SUDHL-2, Ly3, and Ly10 cells inoculated into NOD/SCID mice. When administered at a concentration that did not significantly disturb the development of normal B cells or other hematopoietic lineages of normal mice (Supplementary Fig. 7a), VER155008 exerted a much stronger inhibitory effect on the in vivo growth of RJ-Lym1 cells and SUDHL-2 cells than bortezomib, a new proteasome inhibitor used for the treatment of refractory or relapsing ABC-DLBCL (Fig. 7a)^25^. In contrast, bortezomib exerted a stronger anti-tumor effect on Ly3 and Ly10 cells than VER155008 (Supplementary Fig. 7b). Immunohistochemical analysis of Ki67 and a TUNEL assay on the retrieved RJ-Lym1 samples showed that the HSP70 inhibitor exerted a stronger inhibitory effect on the proliferation and survival of lymphoma cells in vivo than bortezomib under conditions in which both treatments similarly increased Blimp-1 levels (Fig. 7b). As expected, VER155008 also repressed the expression of CD45, CD19, and CD20 more strongly than bortezomib (Fig. 7b), thus indicating that the transcriptional regulatory activity and oncorepressor activity of mutant Blimp-1 proteins were much better restored by the HSP70 inhibitor than the proteasome inhibitor in lymphoma cells growing in vivo. Importantly, the stable knockdown of Blimp-1 in SUDHL-2 cells significantly attenuated the therapeutic effect of VER155008 (Fig. 7c). However, bortezomib indeed significantly raised the XBP1 level (Supplementary Fig. 7c). Altogether, these observations identified this HSP70 inhibitor as a potential therapeutic agent for ABC-DLBCL cases in which Blimp-1 insufficiency is caused by the instability of homogenously expressed, N-terminally misfolded Blimp-1 mutants.

## Discussion

Sumoylation is mainly mediated by two distinct subfamilies of posttranslational modifiers, Sumo-1, and Sumo-2/3. Sumo-2/3 is able to form polymeric chains, and Sumo-1 modifies the substrate in monomeric form or terminates the Sumo-2/3 polymeric chain. Although the substrate acceptor sites for Sumo-1 and Sumo-2/3 can overlap, the functional consequences of these two discrete modifications are potentially different^48^. For example, Sumo-2/3 polymeric chains, but not the monomeric Sumo-1 chains, effectively recruit sumoylation-targeted ubiquitin ligases (STUbLs) (i.e., RNF4) and trigger the ubiquitination and the proteasome-mediated degradation of substrates^42, 49^. In agreement with the results of a previous study indicating that PIAS1 catalyzes the Sumo-1-mediated modification of WT Blimp-1^37^, we eliminated the possibility that PIAS1 destabilizes WT or mutant Blimp-1 proteins. We also provided evidence that nuclear Blimp-1 proteins physically associate with PML, whereby nuclear Blimp-1 proteins are sequentially modified by Sumo-2/3 and Ub. In line with the findings that PML preferentially adds polymeric Sumo-2/3 to misfolded nuclear proteins^42^, we found that PML tends to add more Sumo-2/3 modifications to misfolded mutant Blimp-1 proteins than WT Blimp-1. Nevertheless, as suggested by the high stability of WT Blimp-1, this sumoylation-triggered metabolic pathway occurs at a low rate.

Ultimately, the mutant Blimp-1 proteins were degraded primarily through an Hrd1-mediated cytoplasmic pathway. Hrd1 has been shown to mediate the cytoplasmic sequestration and degradation of WT Blimp-1 in DCs under inflammatory conditions^33^. Nevertheless, we have presented evidence indicating the greater selectivity of Hrd1 for mutant Blimp-1 proteins that harbor N-terminal misfolding mutations in the absence of inflammation. Thus, the majority of WT Blimp-1 can undergo nuclear translocation, after which the metabolism of this protein is completed via the nuclear PML/polymeric sumoylation-triggered pathway described above.

Previous studies have indicated that ER-anchored Hrd1 might directly recognize misfolded proteins^50^. Nevertheless, we found that Hrd1 actually recognizes the WT Blimp-1 aa 127–207 fragment, whose 3D structure is not significantly altered by two ABC-DLBCL-associated destabilizing mutations. Therefore, we explored the mechanisms underlying this specific preference of the Hrd1 pathway for mutant Blimp-1 proteins and thus identified the chaperone HSP70, which recognizes the structurally altered aa 1–130 subregion and escorts mutant Blimp-1 proteins to Hrd1 for recognition. This result differs from the results of previous studies, which have indicated that HSP70 typically guides misfolded proteins for Ub labeling by CHIP^39, 43^. However, in accordance with a previous observation that HSP70 does not facilitate PML recognition of nuclear misfolded substrates^42^, we did not observe an apparent cooperative effect between HSP70 and PML in the metabolism of nuclear Blimp-1 proteins.

Finally, HSP70 inhibitors (such as VER155008) have exhibited promising anti-tumor effects in breast cancer and colon cancer cell lines by inducing tumor cell apoptosis and senescence while sparing normal cells^51, 52^. Specifically, in the treatment of relapsing DLBCL or those refractory to first-line therapy, HSP70 overexpression has been found to be associated with resistance to bortezomib^25, 53^. Theoretically, for those aggressive ABC-DLBCL cases homogenously expressing unstable Blimp-1 mutants^13, 19, 44, 54^, bortezomib not only inhibits NF-κb activity but also stabilizes the unstable Blimp-1 mutants in ABC-DLBCL cells. Nevertheless, we provided evidence that HSP70 inhibition results in a better therapeutic effect by specifically restoring the nuclear accumulation of unstable Blimp-1 mutants that retain their transcriptional regulatory and oncorepressor activities. Thus, our study suggests that HSP70 inhibition may be a promising approach for decreasing the aggressiveness of malignancy, particularly at least in these specific ABC-DLBCL cases carrying unstable Blimp-1 mutants.

## Methods

### Cell lines

Ly3, Ly10, and SUDHL-2 ABC-DLBCL cell lines^13, 26, 27^ were provided by Dr. T Zhao (Nanfang Hospital affiliated to Southern Medical University, China). RJ-Lym1 cells were isolated from a primary sample from a ABC-DLBCL case in the Shanghai Rui-Jin Hospital with informed consent. RJ-Lym1 cells were successfully passaged as xenografts in NOD/SCID mice, but not as an in vitro cultivated cell line. Ly1 and Ly8 lymphoma cell lines were provided by Dr. X-Y Zhou (Fudan University Shanghai Cancer Center). 293T cells, HeLa cells and other tumor cell lines including the U266 multiple myeloma cell line and the Namalwa Burkitt lymphoma cell line were obtained from the cell line bank of the Shanghai Institute of Hematology.

### Cell culture

All lymphoma cell lines were maintained in Isocove’s Modified Dulbecco’s Medium (IMDM) (Gibco, USA) supplemented with 10% fetal bovine serum and 2 mM l-glutamine. 293T cells and HeLa cells were maintained in Dulbecco’s modified Eagle’s medium (DMEM) (Gibco, USA) containing 10% fetal bovine serum.

### Immunoprecipitation

Overall, 3 × 10^7^ cells were washed twice with cold PBS and then extracted with 1 ml of IP-lysis buffer 1 (50 mM pH 7.5 Tris, 150 mM NaCl, 0.5% Triton X-100, 10% glycerol, 2 mM EDTA, freshly added 1 mM PMSF, 20 μM MG132, 10 mM *N*-ethylmaleimide, 20 mM each protease inhibitor cocktail and phosphatase inhibitor cocktail, and 2 mM DTT) at 4 °C for 1 h. For the preparation of nuclear extracts, 3 × 10^7^ 293T cells were extracted with Nuclear-Cytosol Extraction Kit (Applygen Technologies Inc, Beijing, China). Briefly described, the cytosol and membrane fractions were firstly removed with CEB-A and CEB-B, and then the nuclear pellet was dissolved in 2% SDS lysis buffer (50 mM pH 7.5 Tris and 2% SDS), boiled at 100 °C for 20 min and then diluted in IP-lysis buffer 1 by 20-folds. For the co-immunoprecipitation of the endogenous Hrd1, cells were extracted with IP-lysis buffer 2 (100 mM Tris-HCl, 80 mM NaCl, 1 mM EDTA, 5 mM EGTA, 5% glycerol, 2% w/v digitonin, 0.1% Brij 35, freshly added 1 mM PMSF, 20 μM MG132, 10 mM *N*-ethylmaleimide, 20 mM each protease inhibitor cocktail and phosphatase inhibitor cocktail).

For the immunoprecipitation, the cell lysates were then spun at 16,000×*g* for 10 min to remove debris. The collected supernatants were then incubated with 20–50 μl gel preconjugated with anti-FLAG M2 (Sigma, Cat: A2220) or anti-MYC (Biotool, Cat: B23401), or with beads precoated by 10 μg anti-Blimp-1 (Santa Cruz, Cat: sc-66015) or 5–10 μg anti-IgG (CST, Cat: #4097) at 4 °C for 4 h or overnight. The gel or beads were then sequentially washed 5 times with co-IP-lysis buffer. The bound proteins were eluted with 50 μl 2% SDS lysis buffer and boiled at 100 °C for 10 min. The proteins were then analyzed by western blotting.

### Western blotting analysis

For the preparation of total cell lysates, each 5 × 10^6^ cells were washed twice with cold PBS and added with 100 μl lysis buffer (50 mM pH 7.5 Tris and 2% SDS), which was immediately boiled at 100 °C for 10 min. A total of 30–100 μg of protein was loaded into each lane for running on 6–12% SDS-PAGE. Then the proteins were blotted to PVDF membrane (GE Healthcare, Cat: 10600023). Primary antibodies used included anti-flag (rabbit, CST, Cat: #2368 or #14793), anti-Blimp-1 (mouse, Santa Cruz, Cat: sc-66015) (rabbit, CST, Cat: #9115), anti-SUMO-1 (rabbit, Abgent, Cat: AJ1746a, clone ID: Y299), anti-SUMO-2/3 (rabbit, Abgent, Cat: AP1239a, clone ID: RB46399), anti-Ub (HRP conjugate, CST, Cat: #14049), anti-PML (rabbit, Abgent, Cat: #AP51432-100 µL and Santa Cruz, Cat: sc-5621), anti-PIAS1 (rabbit, CST, Cat: #3550), anti-PIAS2 (rabbit, Sigma, Cat: SAB3500483), anti-PIAS3 (rabbit, CST, Cat: #9042), anti-PIAS4 (rabbit, CST, Cat: #4392), anti-CBX4 (rabbit, Abgent, Cat: #AP2514a), anti-RanBP2 (rabbit, Novus, Cat: NB100-93337), anti-Hrd1 (rabbit, Abgent, Cat: #AP2184A and CST, Cat: #14773), anti-HSP70 (rabbit, CST, Cat: #4876), anti-GFP (mouse, Santa Cruz, Cat: sc-9996) (rabbit, CST, Cat: #2956), anti-RNF4 (mouse, Abnova, Cat: H00006047-A01) (rabbit, Sigma, Cat: SAB1100322), anti-SENP1 (rabbit, CST, Cat: #11929), anti-SENP6 (rabbit, Abgent, Cat: AP1224a, clone ID: RB46399), anti-CHIP (rabbit, CST, Cat: #2080), anti-GAPDH (rabbit, CST, Cat: #5174), and anti-β-actin (mouse, Sigma, Product number: A5316, clone AC-74). All these primary antibodies (expect β-actin) were diluted at 1:1000 and β-actin was diluted at 1:10,000 in 5% BSA (Sangon Biotech, Cat: 9048-46-8) in TBST (25 mM Tris-HCl, 140 mM NaCl, and 0.1% v/v Tween-20), then used for staining the blotted membrane at 4 °C overnight. The goat anti-mouse IgG secondary antibody (Calbiochem, Cat: #401215) or goat anti-rabbit IgG secondary antibody (Calbiochem, Cat: #401315) was usually used at a 1:5000 dilution in 5% milk in TBST. The uncropped scans of blots are shown in the Supplementary Fig. 8.

### Immunofluorescence microscopy

Cells that were cultured on coverslips were fixed with 4% paraformaldehyde for 30 min, permeabilized with 0.2% Triton X-100 for 15 min, blocked with 1% BSA and incubated with antibody. Cells were mounted with DAPI-containing medium (Vector Labs) before inspection with microscopy. Primary antibodies to the following proteins were used as indicated in the product information: anti-flag (mouse, Sigma, Cat: F1804, 1:100 dilution) and anti-Blimp-1 (rabbit, CST, Cat: #9115, 1:100 dilution). The secondary antibodies were Texas Red-conjugated anti-mouse IgG (Vector labs, Cat: TI-2000, 1:100 dilution) and Texas Red-conjugated anti-rabbit IgG (Vector labs, Cat: TI-1000, 1:100 dilution).

HeLa cells transfected with the Blimp-1-GFP-overexpressing plasmids were incubated with ER-Tracker Red (Invitrogen, Cat: E34250) according to the manufacturer’s instructions. Briefly described, the adherent cells were rinsed with HBSS (Invitrogen), and 1 μM ER-Tracker Red in pre-warmed HBSS was added to the dish and incubated at 37 °C for 30 min. Then the staining solution was replaced with fresh probe-free culture medium before imaging. 293T cells or HeLa cells were transfected with the plasmids as indicated. Then, four drops of NucBlue Live reagent (Hoechst 33342, Invitrogen, Cat: R37605) were added to 2 ml cell culture and incubated at room temperature for 20 min before imaging. The images were acquired with a Leica TCS SP8 microscope.

### Xenograft mouse model and treatments

The right flanks of NOD/SCID mice (Shanghai Laboratory Animal Center, China) were injected subcutaneously with 1 × 10^7^ RJ-Lym1 cells, SUDHL-2 cells, Ly3 cells and Ly10 cells in 100 μl PBS. When the tumor volumes reached 600–800 mm^3^, mice were randomly divided into 3 groups for treatment with (1) PBS, (2) bortezomib (1 mg kg^−1^), or (3) VER155008 (40 mg kg^−1^) for 21 days.

### Transfection of cDNAs and siRNAs

cDNAs encoding the human proteins fused to flag, GFP, myc, mCherry or YFP at the C- or N-terminus were inserted in plasmids pCMV6-AC-DDK, pEGFP-N1, pcDNA3.0 (-) B, pmCherry-C1 or pEYFP-N1 to produce Blimp-1-flag, Blimp-1-GFP, flag-Blimp-1-GFP (1–206 aa), Blimp-1-GFP (1–130 aa), Blimp-1-GFP (127-206 aa), Hrd1-myc, mCherry-SUMO1, mCherry-SUMO-3, mCherry-Ub, mCherry-PIAS1, mCherry-CBX4, mCherry-PML, mCherry-RNF4, mCherry-HSP70, mCherry-Hrd1, and YFP-Ub. cDNAs encoding SENP1, SENP6, SUMO1, SUMO-2, SUMO-3, PML, RNF4, PIAS1, PIAS2α, PIAS2β, PIAS3, PIAS4, and CBX4 were inserted into pCMV-AC-DDK. DNA plasmids were transfected into cells using Lipofectamine 3000 (Invitrogen).

The siRNAs against *PML*, *CBX4*, *Hrd1*, *HSP70 (HSPA5*, *HSPA8*, and *HSPA9)*, *SUMO-2*, *SUMO-3*, and *SENP1* were purchased from GenePharma (China), and the sense strand sequences were: *PML*, CCCGCAAGACCAACAACAUTT; *CBX4*, GCAAGAGCGGCAAGUACUATT; *Hrd1*, GCAGCUGGUGUUUGGCUUUTT; *HSPA5*, CCAAAGACGCUGGAACUAUTT; *HSPA8*, GGCCAGUAUUGAGAUCGAUTT; *HSPA9*, GCCCUAUCUUACAAUGGAUTT; *SUMO-2*, GCAUACACCACUUAGUAAATT; *SUMO-3*, CAAUGAAACUGACACUCCATT; *SENP1*, CUGCCAUGUAUCUGCAUAUTT. siRNAs were transfected into cells by using Lipofectamine RNAiMAX (Invitrogen) according to the manufacturer’s instructions.

The shRNA targeting human *BLIMP1* was cloned into the hU6-MCS-EGFP vector. The shRNA sequence was: AATTCAAAAACGGCTACAAGACCCTTCCCTA CTCGAG TAGGGAAGGGTCTTGTAGCCG. SUDHL-2 cells in six-well plates were spin infected with lentivirally packaged Blimp-1 shRNA and negative control GFP shRNA for 90 min. The cells were incubated at 37 °C for 4 days. Then, cells were harvested and FACS purified (BD FACSAria III) to obtain Blimp-1 shRNA- or negative control GFP shRNA-transduced (GFP^+^) SUDHL-2 cells.

### Immunoprecipitation assay and LC-MS/MS analysis

For the immunoprecipitation (IP) assay, cells were lysed with 2% SDS lysis buffer (50 mM pH 7.5 Tris and 2% SDS) and boiled for 15–20 min. The lysates were diluted 20-fold in co-IP-lysis buffer 1 (50 mM pH 7.5 Tris, 150 mM NaCl, 0.5% Triton X-100, 10% glycerol, 2 mM EDTA, freshly added 1 mM PMSF, 20 μM MG132, 10 mM *N*-ethylmaleimide, 20 mM each protease inhibitor cocktail and phosphatase inhibitor cocktail, and 2 mM DTT), and Blimp-1-flag proteins were immunoprecipitated with anti-FLAG M2 gels (Sigma, Cat: A2220) and analyzed for SUMO-1, SUMO-2/3, or Ub modification.

For LC-MS/MS (Liquid Chromatography-Mass Spectrometry/Mass Spectrometry) analysis, 293T cells transfected with WT or mutant Blimp-1-flag-expressing plasmids were treated with or without MG132 for 12 h. These cells were lysed with co-IP-lysis buffer 2 (100 mM Tris-HCl, 80 mM NaCl, 1 mM EDTA, 5 mM EGTA, 5% glycerol, 2% w/v digitonin, 0.1% Brij 35, freshly added 1 mM PMSF, 20 μM MG132, 10mM *N*-ethylmaleimide, 20 mM each protease inhibitor cocktail, and phosphatase inhibitor cocktail), and then proteins were co-immunoprecipitated with anti-FLAG M2 beads and further analyzed by LC-MS/MS.

### Molecular simulation

The N-terminal Blimp-1 structure was reconstructed with the crystal structure in the PDB database (entry: 3DAL, aa 38–223, which was determined by X-ray crystallography at 1.65 Å resolution), with the Modeller 9.16 program^55^ used to extrapolate the missing loop regions, such as aa 69–76, and the lowest energy structure (DOPE score: −166,773.7) and for the follow-up mutation and molecular simulation. Molecular dynamic simulations were carried out with a ff03 force field^56^. These protein models were protonated at pH 7 and solvated within an octahedron water box of TIP3P^57^ with a water thickness above 10 Å from the protein surface. Sodium ions were added for charge neutralization. The solvated systems were subjected to 10,000-step minimization with the steepest descent method for the first 1000 cycles and then the conjugate gradient method. The systems were then gradually heated from 0 to 300 K for 50 ps, as controlled by the Langevin constraint with a collision frequency of 2 ps^−1^. Next, the constant pressure and temperature (NPT) ensemble, with the Berendsen algorithm used to set the barostat and 300 K thermo bath, was applied for 50 ps equilibrium. Finally, 50 ns production simulations were carried out under the NPT ensemble without any restraint. In the simulation, the timestep was set as 2 fs, and the van der Waals cutoff was set at 10 Å. The Particle Mesh Ewald method^58^ was used to calculate long-range electrostatic interactions, and structural snapshots were flushed every 500 steps. All MD simulations were performed using the parallel version of pmemd implemented in the AMBER 12^59^ suite, and each system was simulated three times individually. Root mean-square fluctuation (RMSF) values of the alpha carbon atom were calculated, and root mean-square deviation (RMSD)-based clustering was completed with an average linkage clustering algorithm. Representative structures were extracted to describe the conformational changes. The clustering and the hydrophobic surface area were calculated by cpptraj in AMBER 12.

### Quantitative RT-PCR analysis

Total RNA was extracted using Trizol (Invitrogen). Reverse transcription of total RNA was performed using an RT reagent kit (TOYOBO). Quantitative real-time PCR was carried out via SYBR Green PCR (Takara). Relative expression levels were calculated using the ΔΔCT method using human 18S rRNA as an internal reference. The sequences of primer pairs used are listed in the Supplementary Table 4.

### Flow cytometric analysis

Overall, 1 × 10^6^ cells were suspended with 100 μl PBS and stained with BV-421-anti-CD138 (BioLegend, Cat: 356515, clone: MI15, 1:20 dilution), BV-785-anti-CD45 (BioLegend, Cat: 304047, clone: HI30, 1:20 dilution), APC-anti-CD19 (eBioscience, Cat: A18615, clone: HIB19, 1:20 dilution), BV-421-anti-Ki67 (BD, Cat: 565929, clone: B56, 1:20 dilution), APC-anti-Annexin V (BioLegend, Cat: 640920, 1:20 dilution), and 7-AAD (BD, Cat: 559925, 1:20 dilution) by following the manufacturer’s instructions. All of the flow cytometric analyses were performed on an LSR II Fortessa cytometer (BD Biosystems), and the data were analyzed using FlowJo software.

### Luciferase reporter assay

The human CIITA promoter region spanning nt −545 to +123 and encompassing a Blimp-1 binding site at position −180 nt directed luciferase expression in the CIITA-Luc reporter construct. For luciferase reporter assays, 293T cells or 293-Hrd1-KO cells were co-transfected with 0.25 μg, 1 μg, or 1.5 μg pCMV6-AC-Blimp-1-flag vector, 1.5 μg CIITA-Luc reporter construct, and 0.1 μg TK-RL Renilla reporter. Cells were collected 48 h after transfection, and the Dual Luciferase Reporter Assay (Promega, Cat: E1910) was performed according to the manufacturer’s instructions. Briefly described, 293T cells were rinsed with PBS, added with PLB lysis buffer and gently rocked at room temperature for 15 min before transferring the cell lysates to a new 1.5 ml tube for sequentially measuring the firefly luciferase activity and *Renilla* luciferase activity.

### Generation of *Hrd1*^−/−^ 293T cells and *PML*^−/−^ HeLa cells

We used the CRISPR/Cas9 system to establish the *Hrd1* ^−/−^ 293T cell line. Two guide RNA sequences targeting the *Hrd1* locus were designed: GTTCCGCACGGCAGTGATGATGG and CGTACCAGGAACGTTCCAGAAGG. 293T cells were transfected with the plasmids expressing the guide RNAs and Cas9 by electroporation. Then, the cells were dissociated into single cells and placed in ten 96-well plates with 1–5 cells per well. Fourteen days later, genomic DNA was extracted from the cells to screen for the correct clones. The *PML* ^−/−^ HeLa cell line was a gift from Dr. Meng GY from the Shanghai Institute of Hematology.

### IHC and TUNEL assays

For immunohistochemistry (IHC) assay, fixed slides containing samples from NOD/SCID mice were incubated in 3% H_2_O_2_ for 10 min to suppress endogenous peroxidase activity, washed in PBS at room temperature for 30 min and subsequently washed in BSA for 20 min. Then, the slides were incubated with primary antibodies against Ki67 (rabbit, Abcam, Cat: ab16667, 1:100 dilution), Blimp-1 (rabbit, Abcam, Cat: ab198287, 1:500 dilution), CD19 (rabbit, Abcam, Cat: ab134114, 1:500 dilution), CD20 (rabbit, Abcam, Cat: ab78237, 1:250 dilution), CD45 (rabbit, Santa Cruz, Cat: sc-25590, 1:250 dilution), XBP1 (rabbit, Abcam, Cat: ab109221, 1:500 dilution), CD10 (rabbit, Abcam, Cat: ab126593, 1:500 dilution), BCL6 (rabbit, Abcam, Cat: ab183308, 1:100 dilution), or IRF4 (MUM1) (rabbit, Abcam, Cat: ab133590, 1:500 dilution) overnight. After being washed for 15 min, the slides were incubated with biotin-conjugated secondary antibodies for 30 min and further incubated with a solution of DAB (Boster, Wuhan, China). The tissue-containing slides were examined under a microscope to ensure appropriate staining. The final steps were counterstaining, dehydrating, clearing, and mounting.

For the terminal deoxyribonuleotidyl transferase (TdT)-mediated dUTP nick-end labeling (TUNEL) assay, frozen sections were evaluated with a recombinant terminal transferase kit (Roche, Cat: 11684795910) and then were staining with ProLong™ Gold Antifade Mountant with DAPI (Invitrogen, Cat: P36931) according to the manufacturer’s instructions. Cells with green fluorescence were considered apoptotic.

### In vitro SUMOylation assay

Flag-Blimp1-MYC was expressed in 293T cells and purified with anti-FLAG M2 (Sigma, Cat: A2220). In brief, 3 × 10^7^ cell were extracted with 1 ml of co-IP-lysis buffer (50 mM pH 7.5 Tris, 150 mM NaCl, 0.5% Triton X-100, 10% glycerol, 2 mM EDTA, 1 mM PMSF, 20 mM each protease inhibitor cocktail, and phosphatase inhibitor cocktail, and 2 mM DTT). Supernatants were then incubated with 60 μl preconjugated anti-FLAG M2 (Sigma) at 4 °C overnight. The beads were sequentially washed 5 times with co-IP-lysis buffer, and the bound proteins were eluted in 60 μl elution buffer containing 0.15 mg ml^−1^ 3xFLAG peptide (APExBIO). Reagents for the in vitro SUMOylation reactions were purchased from Boston Biochem. In vitro SUMOylation assays were performed at 37 °C for 75 min in a 20 μl reaction system containing purified Flag-Blimp1-MYC (150 ng), Ubc9, SUMO-2, and SUMO E1 enzyme. The reaction mixtures were denatured by the addition of 20 μl IP-lysis buffer containing 2% SDS and 50 mM DTT and heated at 90 °C for 5 min. One aliquot of the heated reaction mixture was saved for western blot analysis, and the remaining aliquots were diluted 20-fold in IP-lysis buffer without SDS. Flag-Blimp1-MYC was immunoprecipitated by 10 μl of anti-MYC affinity gel (Biotool) and analyzed for SUMO-2/3 modification using anti-SUMO-2/3 antibodies.

### Statistical analysis

The results are expressed as the mean ± SD (standard deviations). All experiments were performed at least three times and were analyzed by one-way ANOVA using GraphPad Prism 6.0 software. *P* values <0.05 were considered statistically significant.

### Data availability

The data that support the findings of this study are available from the corresponding author upon reasonable request.

## Electronic supplementary material

Supplementary Information
