# Supplementary material for: HSP70-Hrd1 axis precludes the oncorepressor potential of N-terminal misfolded Blimp-1s in lymphoma cells
Source: Nat Commun. 2017 Aug 25;8:363. doi: 10.1038/s41467-017-00476-w (PMC5572455; doi:10.1038/s41467-017-00476-w)
Supplement: Supplementary file 1 — Supplementary Information [file 41467_2017_476_MOESM1_ESM.pdf]

Description of Supplementary Files

File name: Supplementary Information
Description: Supplementary figures and supplementary tables.

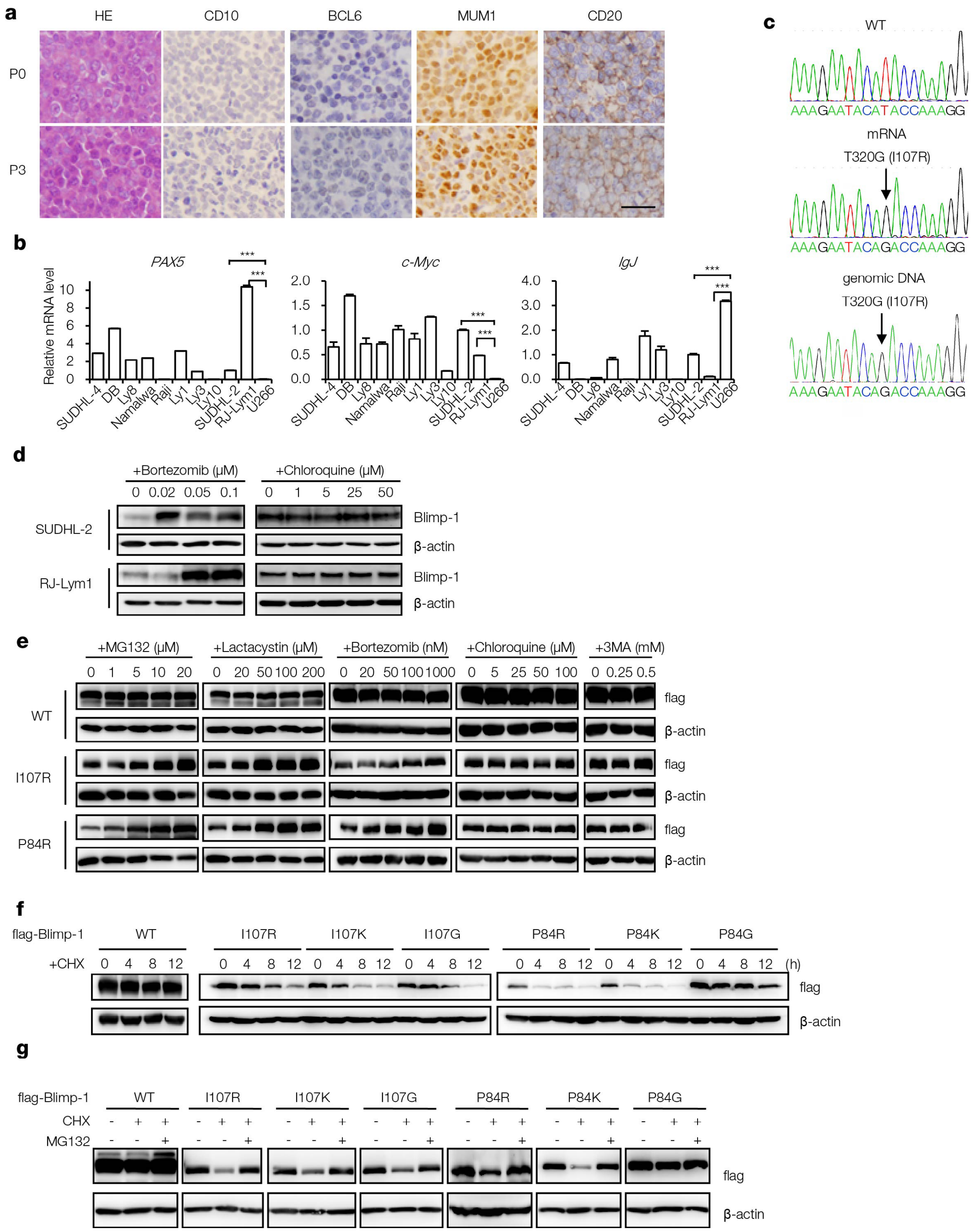

Supplementary Figure 1.

N-terminal misfolding mutations render Blimp-1 susceptible to proteasome-mediated degradation; related to Figure 1. (**a**) IHC staining to monitor the expression of CD20, BCL6 and MUM1 in primary tissue (P0) or from the passage 3 (P3) xenografts of RJ-Lym1 cells in NOD/SCID mice. Scale bar: 10 μm. (**b**) The

mRNA levels of Blimp-1-regulated genes, including *PAX5*, *c-Myc* and *IgJ,* in the indicated lymphoma cells were measured by semi-quantitative RT-PCR. (**c**) Sequencing data for the genomic DNA and mRNA encoding *Blimp-1* indicate that the Blimp-1 mutant (I107R) is homogenously expressed in RJ-Lym1 cells. (**d**) The restorative effects of the proteasome inhibitor bortezomib or the lysosome inhibitor chloroquine on Blimp-1 protein levels in SUDHL-2 or U266 cells were measured by western blotting. (**e**) The effects of the proteasome inhibitors MG132, lactacystin and bortezomib, the lysosome inhibitor chloroquine and the autophagy inhibitor 3MA on the protein levels of WT or mutant Blimp-1 proteins expressed in 293T cells. (**f**) The half-life of the flag-tagged WT or indicated mutant Blimp-1 proteins expressed in 293T cells, as indicated. (**g**) 293T cells were transfected with plasmids encoding flag-tagged WT or mutant Blimp-1 protein and treated with the chemical agents or left untreated, as indicated. Whole cell lysates were analyzed by western blotting. Data are expressed as the mean ± SD. ***P ≤ 0.01, *P ≤0.05.

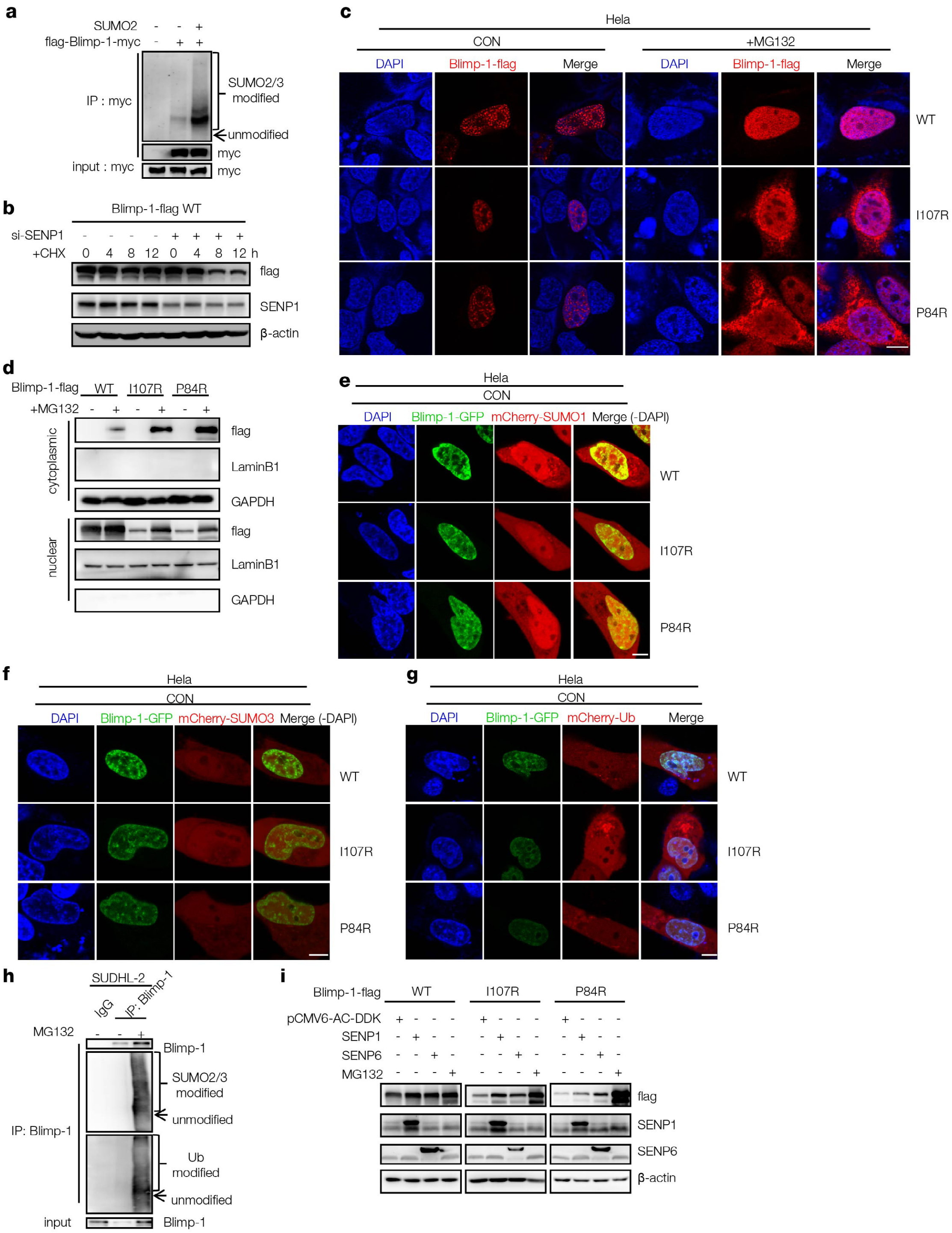

Supplementary Figure 2.

WT and mutant Blimp-1 proteins are differentially metabolized; related to Figure 2. (**a**) Flag- and myc-tagged WT Blimp-1 was immunoprecipitated with anti-flag gel from 293T cells and incubated in the presence of Sumo E1 ligase and Ubc9

with or without purified Sumo2 for 75 min. Sumo2 modification was monitored by western blotting with an anti-Sumo2/3 antibody. (**b**) 293T cells transfected with a flag-tagged Blimp-1-expressing plasmid were treated with control or si-SENP1. The Blimp-1 level was measured by western blotting at different time points after the addition of CHX. (**c**) HeLa cells were transfected with constructs encoding flag-tagged WT or mutant Blimp-1 proteins, treated with MG132 (20 μM) or left untreated for 12 h, stained with an anti-flag antibody (red) and DAPI (blue), and then observed under a confocal microscope. Scale bar: 10 μm. (**d**) 293T cells expressing WT or mutant Blimp-1 proteins were treated with MG132 or left untreated for 24 h, and extracts were collected for subcellular fractionation. The relative cytoplasmic and nuclear distribution of Blimp-1 proteins was then estimated by western blotting. (**e-g**) HeLa cells co-expressing GFP-tagged WT or mutant Blimp-1 proteins with mCherry-Sumo1 (**e**), mCherry-Sumo3 (**f**), or mCherry-Ub (**g**) were observed under a confocal microscope. Scale bar: 7.5 μm. (**h**) SUDHL-2 cells were treated with MG132 or left untreated for 24 h, and cell lysates were immunoprecipitated with an anti-Blimp-1 antibody and immunoblotted with anti-Sumo2/3 or anti-Ub antibodies. (**i**) WT- or mutant Blimp-1-expressing plasmids were co-transfected with empty plasmid (pcDNA3.0 (-) B) or SENP1- or SENP6-expressing plasmids into 293T cells. Total lysates were analyzed by western blotting.

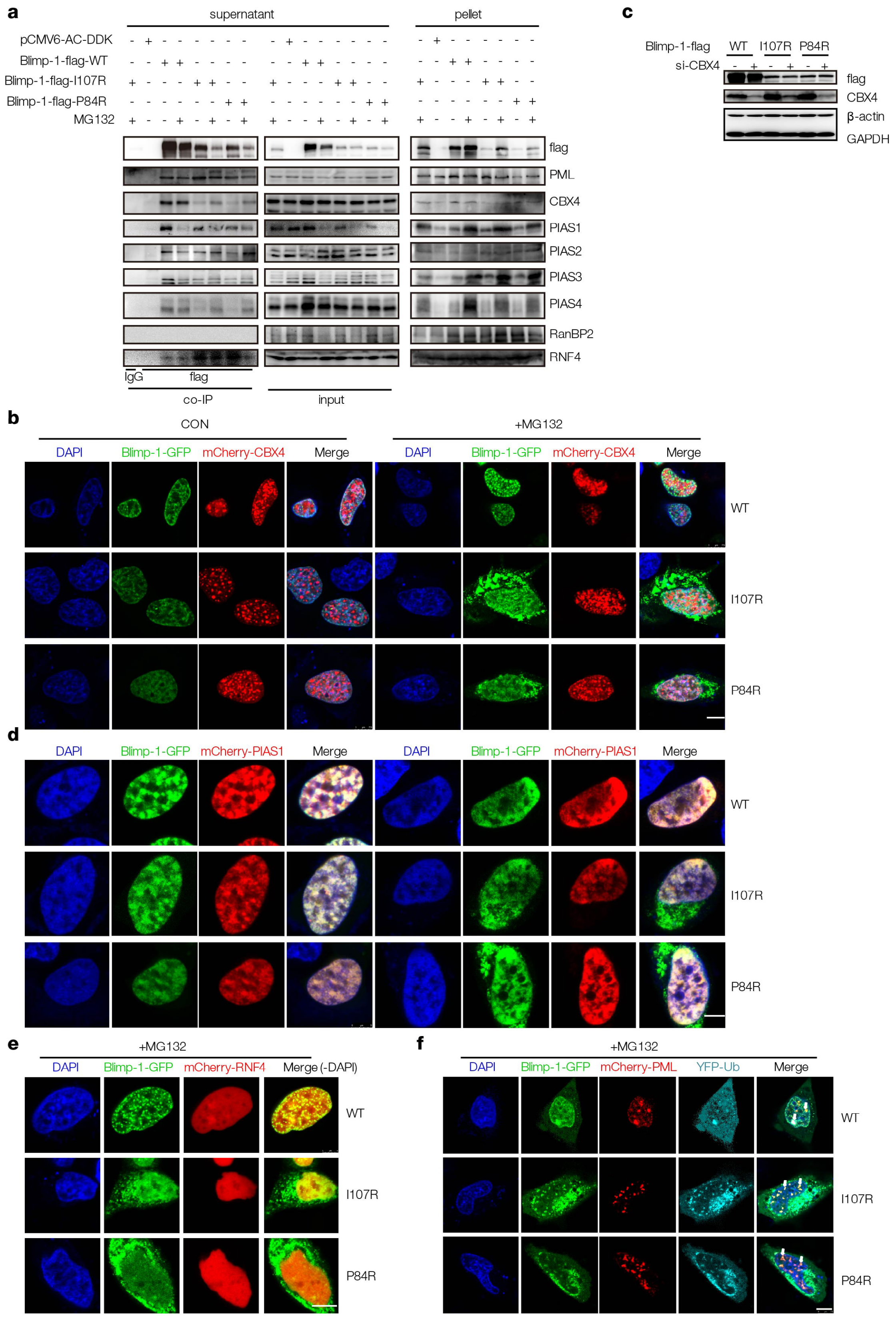

Supplementary Figure 3.

The nuclear metabolism of WT and mutant Blimp-1 proteins; related to Figure 3.

(a) Flag-tagged WT or mutant Blimp-1 proteins were overexpressed in 293T cells, which were treated with MG132 (20 μM) or left untreated for 12 h. Whole cell extracts were co-immunoprecipitated with control IgG or a flag antibody and then immunoblotted with antibodies as indicated. The distribution of the proteins of interest within the pellet was determined by western blotting after dissolution in 2% SDS lysing buffer. (**b**) HeLa cells co-expressing GFP-tagged WT or mutant Blimp-1 protein with mCherry-CBX4 were treated with MG132 or left untreated for 12 h and observed under a confocal microscope. Scale bar: 5 μm. (**c**) Western blot assay to determine the expression of flag-tagged Blimp-1 proteins in 293T cells with or without CBX4 knockdown. (**d**) HeLa cells co-expressing GFP-tagged WT or mutant Blimp-1 protein with mCherry-PIAS1 were treated with MG132 or left untreated for 12 h and observed under a confocal microscope. Scale bar: 5 μm. (**e**) HeLa cells co-expressing mCherry-RNF4 with GFP-tagged WT or mutant Blimp-1 protein were treated with MG132 for 12 h and observed under a confocal microscope. Scale bar: 7.5 μm. (**f**) HeLa cells co-expressing mCherry-PML, YFP-Ub and GFP-tagged WT or mutant Blimp-1 protein were treated with MG132 for 12 h and observed under a confocal microscope. Scale bar: 7.5 μm.

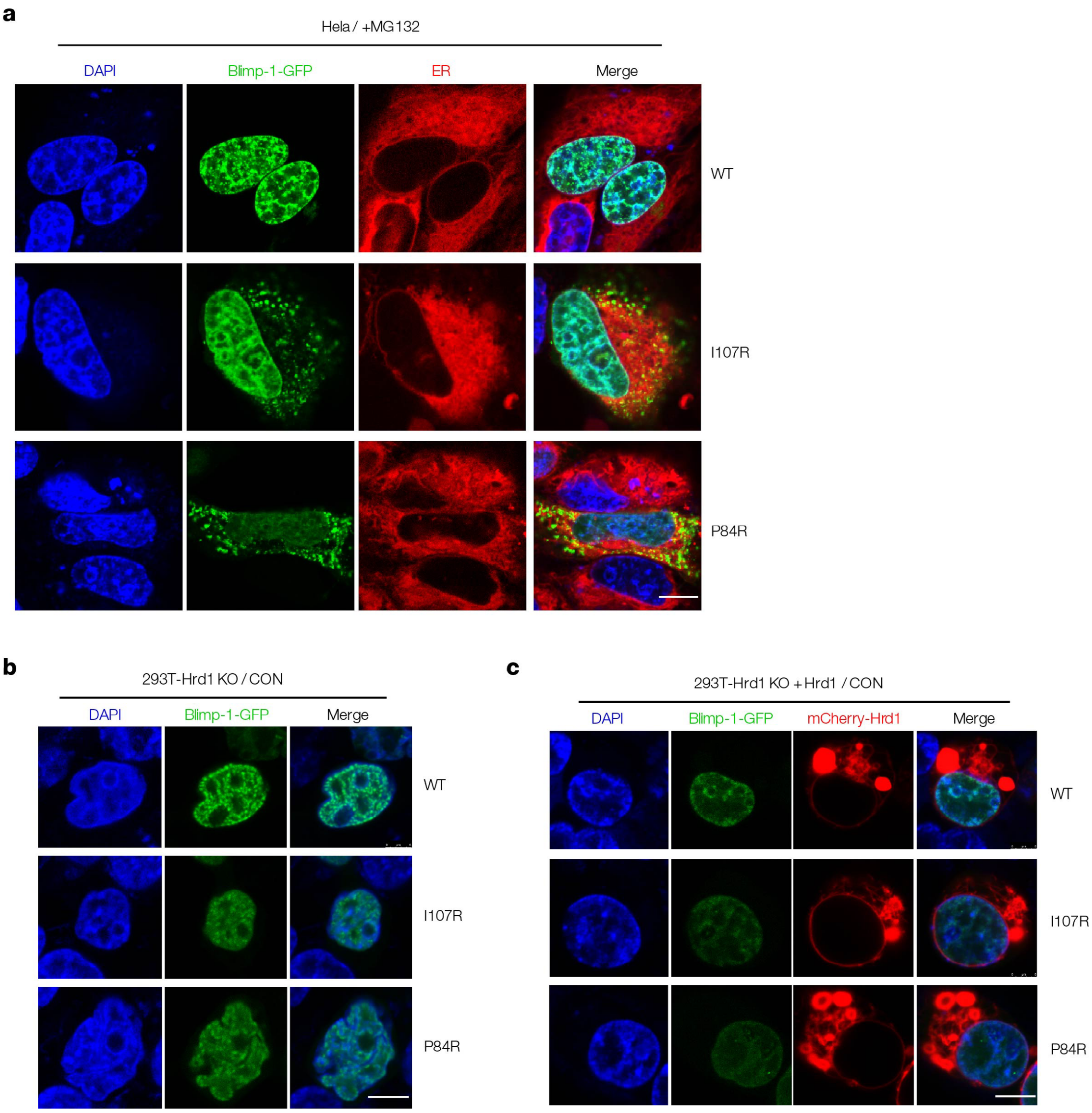

Supplementary Figure 4.

Cytoplasmic degradation of mutant Blimp-1 proteins; related to Figure 4. (a) HeLa cells expressing GFP-tagged WT or mutant Blimp-1 protein were treated with MG132 for 12 h, stained with ER-Tracker Red and NucBlue Live ReadyProbes, and observed under a confocal microscope. Scale bar: 10 µm. (**b**) *Hrd1*$^{-/-}$ 293T cells expressing GFP-tagged WT or mutant Blimp-1 protein were observed under a confocal microscope. Scale bar: 7.5 µm. (**c**) *Hrd1*$^{-/-}$ 293T cells co-expressing GFP-tagged WT or mutant Blimp-1s with mCherry-Hrd1 were observed under a confocal microscope. Scale bar: 7.5 µm.

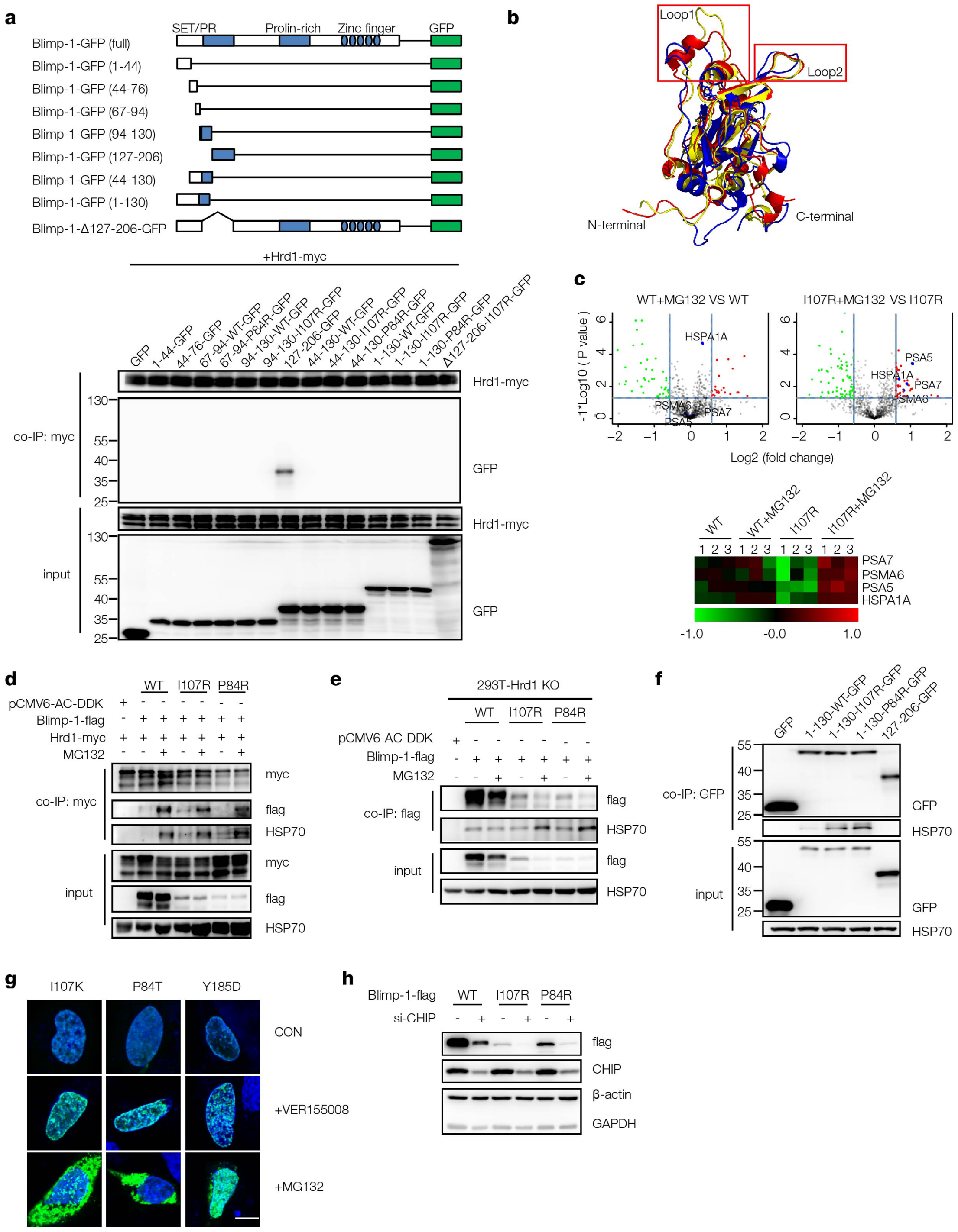

Supplementary Figure 5.

HSP70 promotes the cytoplasmic degradation of mutant Blimp-1 proteins; related to Figure 5. (a) The N-terminus of Blimp-1 (aa 1-206) was divided into different fragments partly according to its secondary structure and fused to GFP at its N-terminus (upper panel). The fusion proteins were co-expressed with Hrd1-myc in

293T cells. The lysates were co-immunoprecipitated with anti-myc antibody and then immunoblotted with anti- GFP antibody (bottom panel). (**b**) Alignment of the representative structures of WT Blimp-1 (yellow) and the P84R (red) and I107R (blue) mutants (simulated). The significantly altered regions in mutants are boxed in red. (**c**) Mass spectrometric analysis of the interacting partners of mutant Blimp-1 proteins during proteasome-mediated degradation. The relative abundance of the candidate proteins associated with WT Blimp-1 (left) or the Blimp-1 I107R mutant (right) isolated before or after MG132 treatment is shown on the volcano plot (the locations of HSP70 and the proteasome subunits are indicated by blue dots) and the heatmap. (**d**) 293T cells co-expressing Hrd1-myc and flag-tagged WT or mutant Blimp-1 proteins were treated with MG132 or left untreated for 12 h. The lysates were then co-immunoprecipitated with anti-myc antibody and immunoblotted with antibodies against the indicated proteins. (**e**) *Hrd1*$^{-/-}$ 293T cells overexpressing flag-tagged WT or mutant Blimp-1 were treated with MG132 or left untreated for 12 h. The interaction between Blimp-1-flag and endogenous HSP70 were analyzed with a co-IP assay. (**f**) The N-terminus of Blimp-1 (aa 1-206) was divided into the aa 1-130 fragment containing P48R or I107R and the aa 127-207 fragment and then fused to GFP at its N-terminus. The fusion proteins were expressed in 293T cells. Lysates were co-immunoprecipitated with anti-GFP antibody and then immunoblotted with antibodies against GFP or HSP70. (**g**) HeLa cells overexpressing GFP-tagged mutant Blimp-1 proteins as indicated were treated with VER155008 or MG132 for 12 h and then observed under a confocal microscope. Scale bar: 10 μm. (**h**) Western blot assay of the expression of flag-tagged Blimp-1 proteins in 293T cells with or without CHIP knockdown.

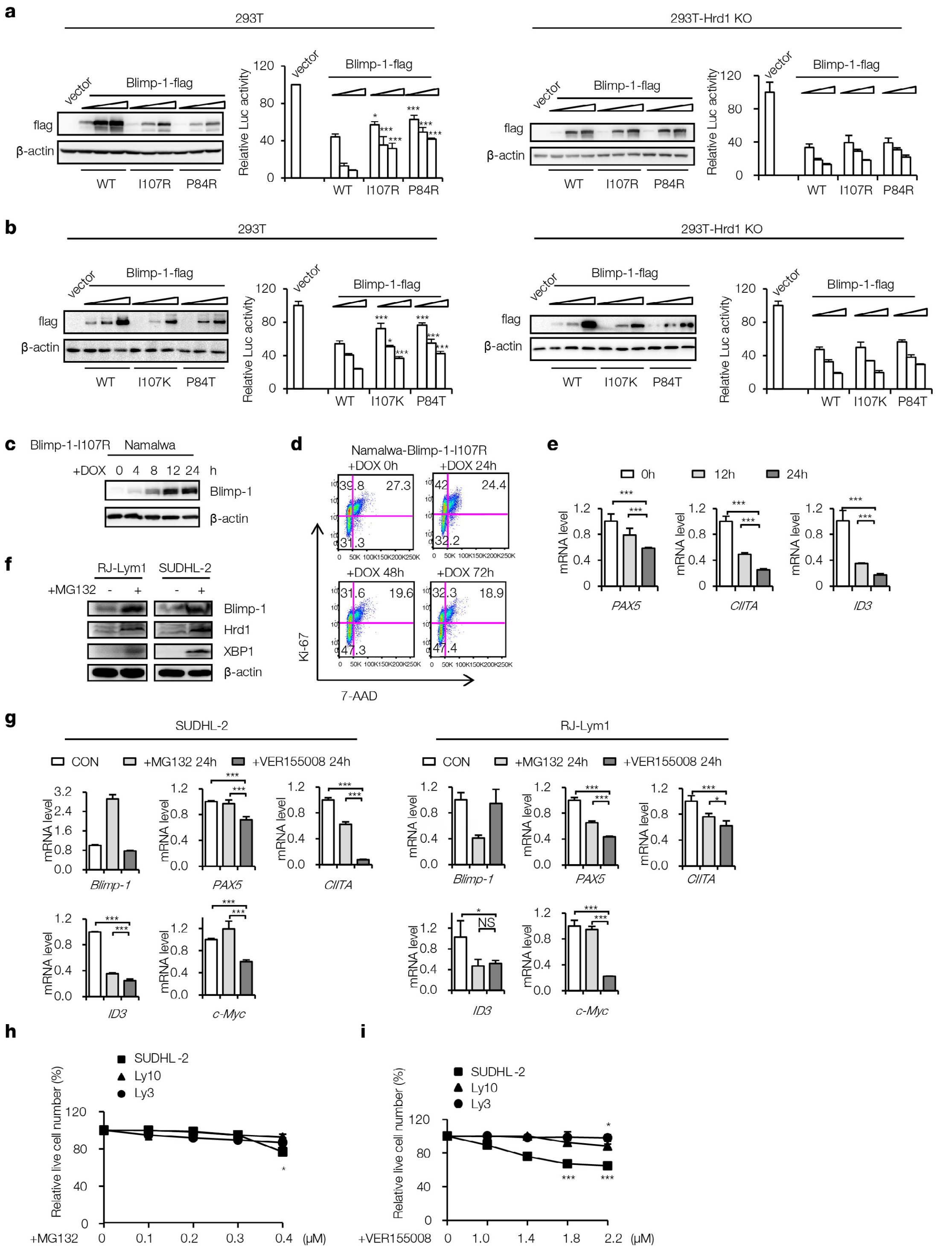

Supplementary Figure 6.

HSP70 inhibition restores nuclear levels of mutant Blimp-1; related to Figure 6.

(**a**) WT and P84/I107R mutant Blimp-1 protein-encoding plasmids were cotransfected with a luciferase reporter construct driven by the human *CIITA* promoter (the nt -545

to +123 region, which encompasses a consensus Blimp-1 binding site at position -180 nt) into 293T cells or 293T-Hrd1 KO cells. The transcriptional repressing activities of Blimp-1 proteins were evaluated with a luciferase assay. The expression of Blimp-1 protein was monitored by western blotting (left panels). P values indicate differences between the mutant groups and the WT group when the same amount of plasmid was used in the transfection. (**b**) The transcriptional regulatory activities of the P84T and I107K mutants were measured as in (**a**). (**c**) Dox-induced expression of the Blimp-1 I107R mutant in Namalwa lymphoma cells. (**d-e**) The cell cycle evaluation (**d**) and target gene expression analysis (**e**) of Namalwa lymphoma cells after I107R mutant induction. (**f**) Western blot assay for XBP-1 and Hrd1 expression after MG132 treatment in RJ-Lym1 cells and SUDHL-2 cells. (**g**) RT-PCR assay of the expression of Blimp-1 target genes in lymphoma cells treated with MG132 or VER155008 for 24 h. (**h-i**) The number of viable SUDHL-2, OCI-Ly3, and OCI-Ly10 cells was measured with a CCK-8 assay after cells were treated with different concentrations of MG132 (h) or VER155008 (i) for 24 h. Data are expressed as the mean ± SD. ***$P \leq 0.01$, *$P \leq 0.05$，NS (no significance).

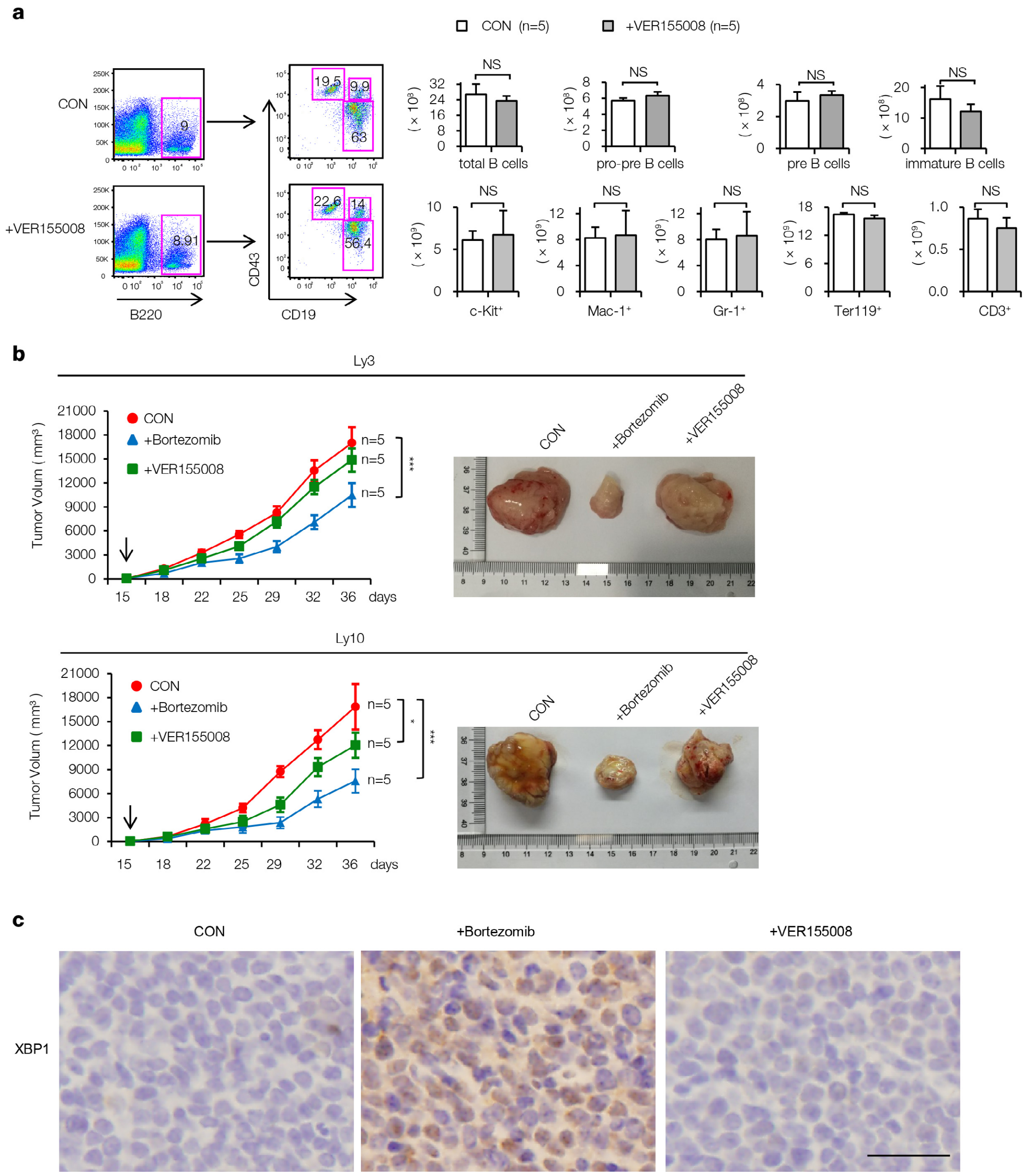

Supplementary Figure 7.

An HSP70 inhibitor suppresses the *in vivo* growth of ABC-DLBCL carrying unstable Blimp-1 mutants; related to Figure 7. (**a**) C57BL/6 mice in each cohort were treated with PBS (CON) or VER155008 (40 mg kg^{-1}) twice weekly for one week. Bone marrow cells were analyzed by flow cytometry. There were no differences in the differentiation of B cells (upper panel) or other hematopoietic lineages (bottom panel) between the CON and VER155008 treatment groups. (**b**)

Mice in each cohort were treated with PBS (CON), bortezomib (1 mg kg^{-1}) or VER155008 (40 mg kg^{-1}) twice every week for 3 weeks. Tumor volumes were measured every 3-4 days. Treatment began on the day indicated with the arrow. (**c**) Mice in each cohort were treated with PBS (CON), bortezomib (1 mg kg^{-1}) or VER155008 (40 mg kg^{-1}) twice weekly. IHC staining was used to examine the expression of XBP1 in tumor specimens. Data are expressed as the mean ± SD. ***P ≤ 0.01, *P ≤ 0.05，NS (no significance).

Fig 1a

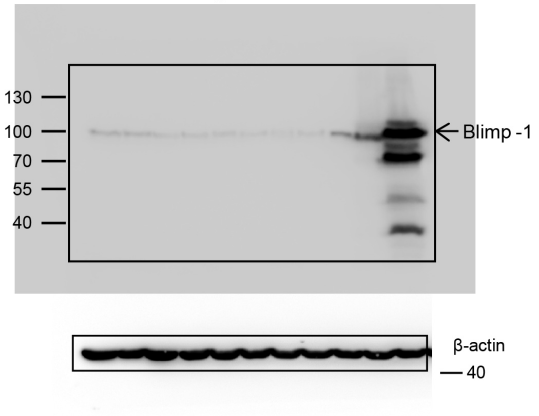

Fig 1d

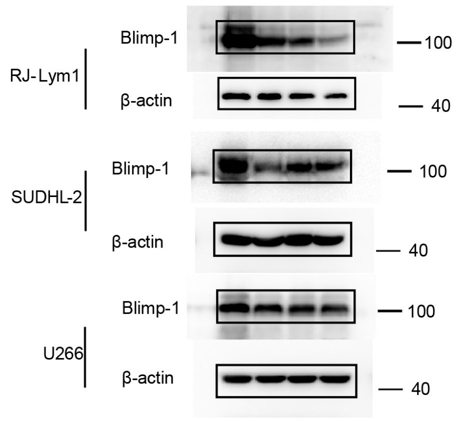

Fig 1e

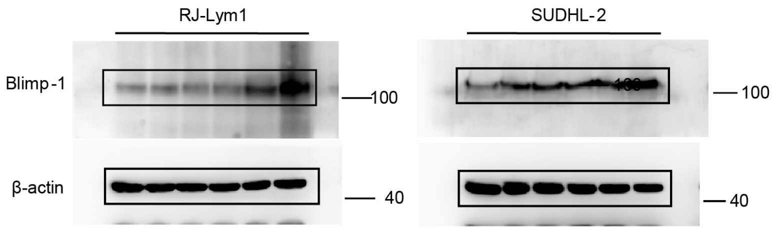

Fig 1f

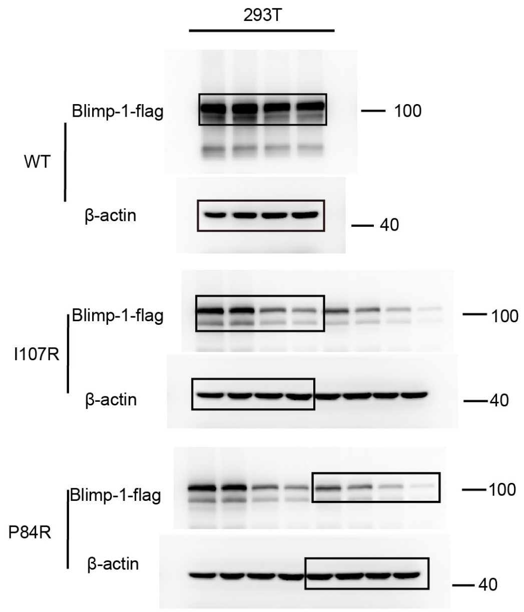

Fig 1j

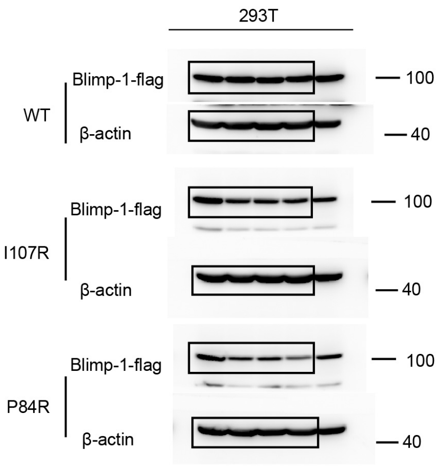

Fig 1g

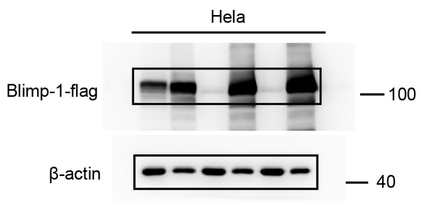

Fig 2a

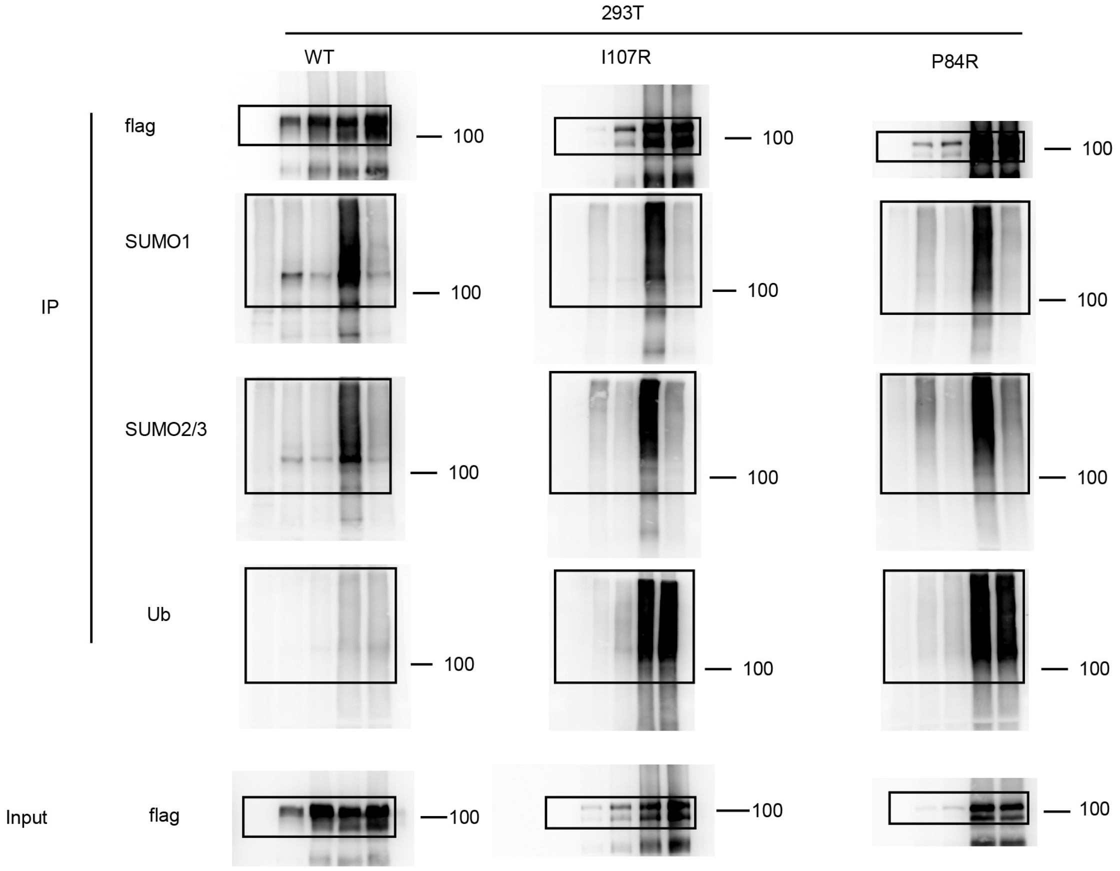

Fig 3a

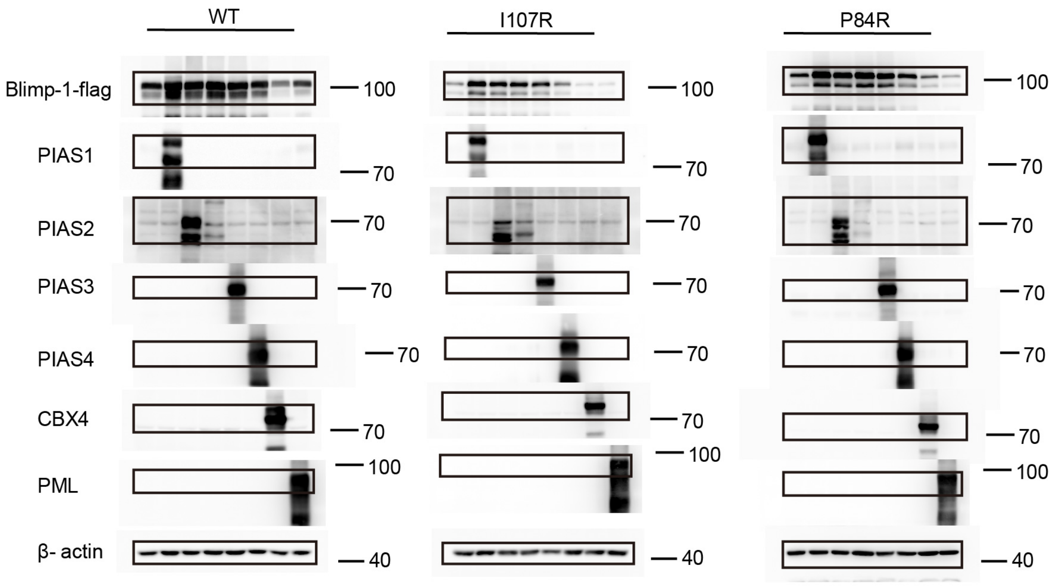
WT
I107R
P84R
Blimp-1-flag
PIAS1
PIAS2
PIAS3
PIAS4
CBX4
PML
β- actin
100
70
40

Fig 3b

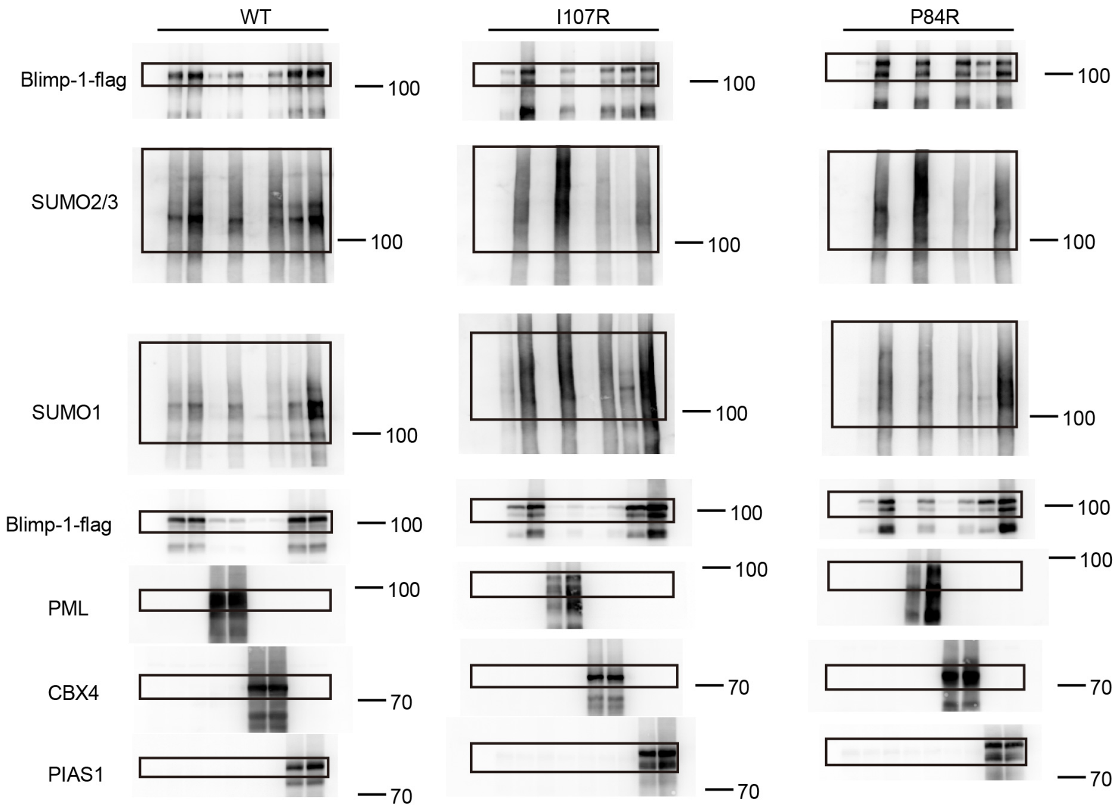
WT
I107R
P84R
Blimp-1-flag
SUMO2/3
SUMO1
Blimp-1-flag
PML
CBX4
PIAS1
100
70

Fig 3d

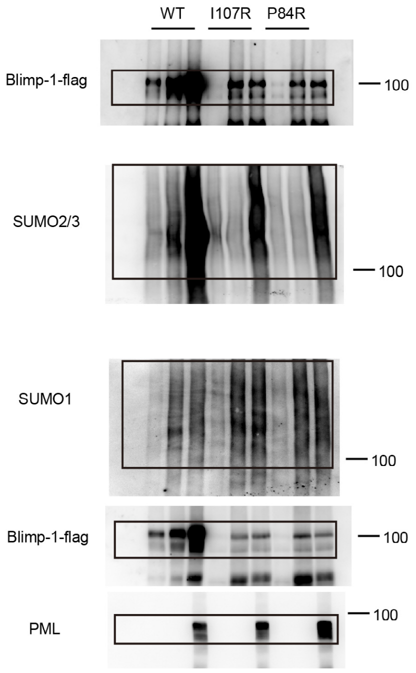

Fig 3f

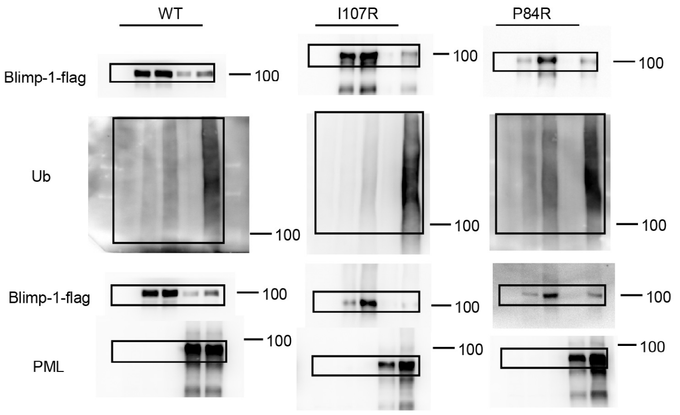

Fig 3g

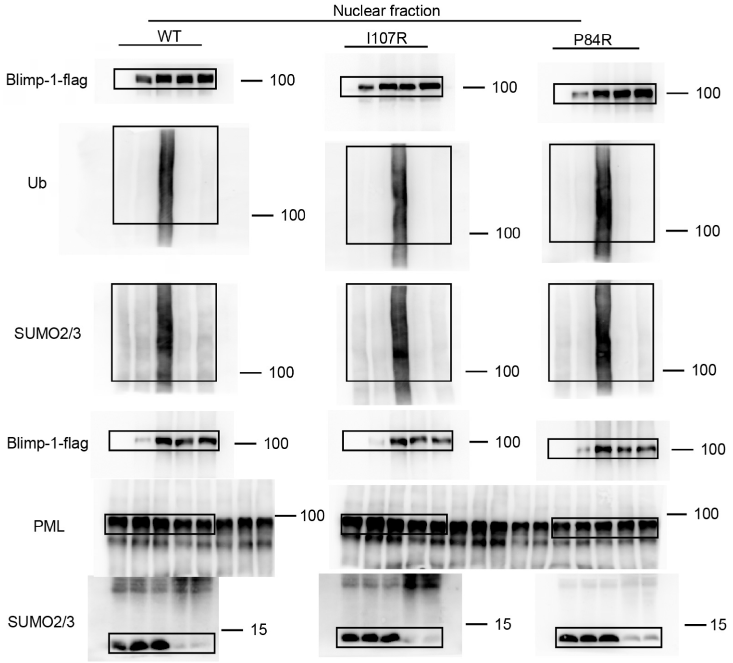

Fig 3e

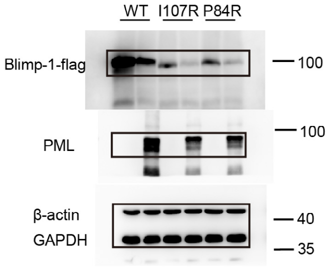

Fig 4d

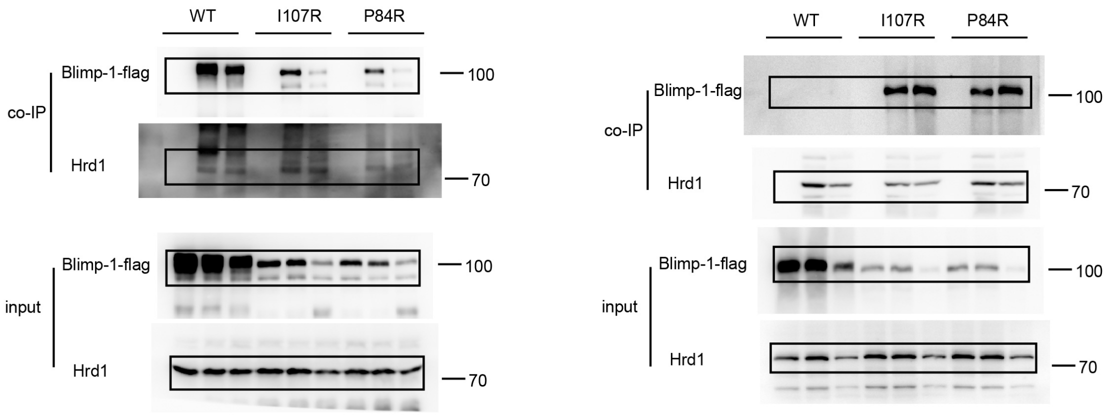
WT
I107R
P84R
co-IP
Blimp-1-flag
Hrd1
input
Blimp-1-flag
Hrd1
100
70
WT
I107R
P84R
co-IP
Blimp-1-flag
Hrd1
input
Blimp-1-flag
Hrd1
100
70

Fig 4e

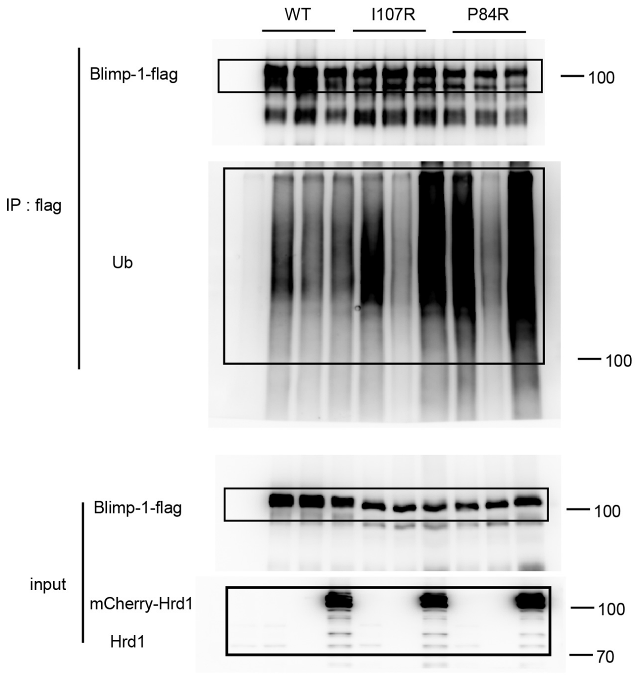
WT
I107R
P84R
IP : flag
Blimp-1-flag
Ub
input
Blimp-1-flag
mCherry-Hrd1
Hrd1
100
70

Fig 5a

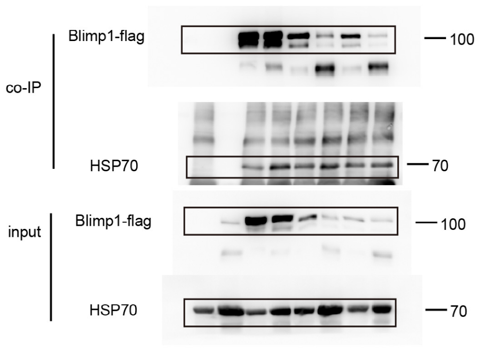

Fig 5d

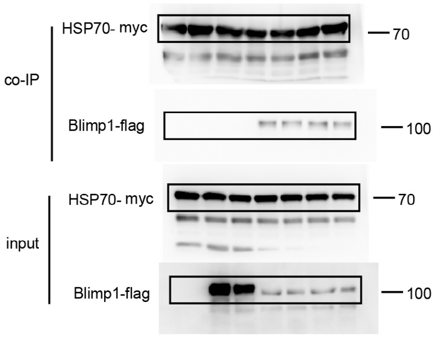

Fig 5c

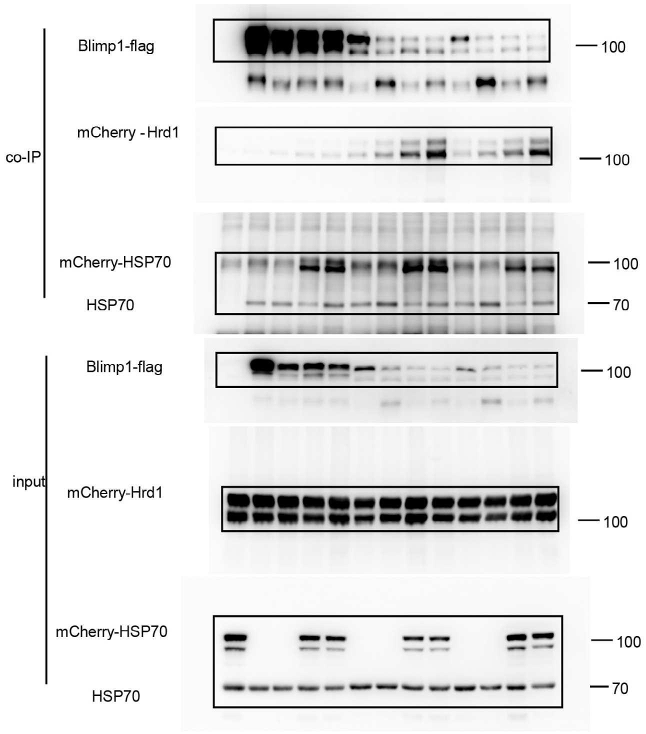

Fig 5e

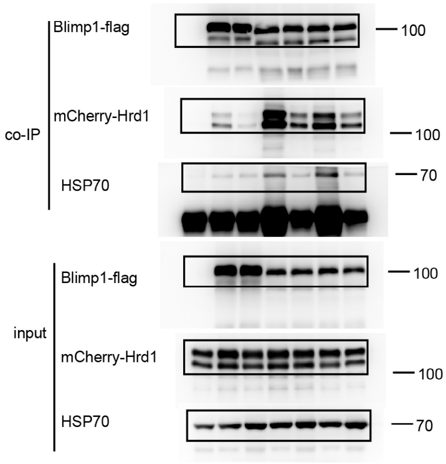

Fig 5g

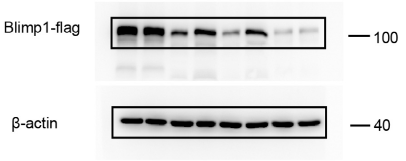

Fig 6a

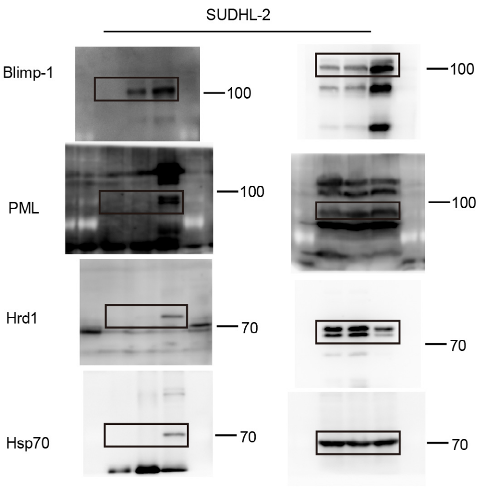

Fig 6b

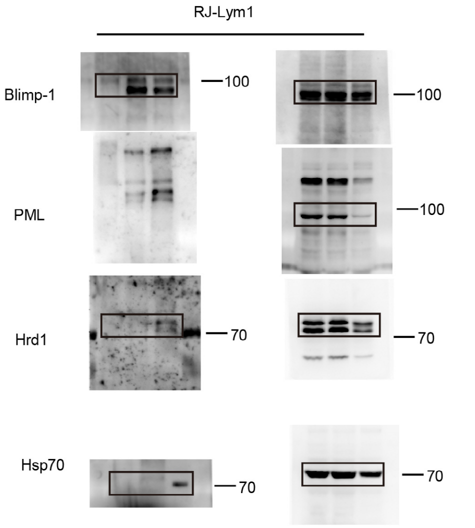

Fig 6c

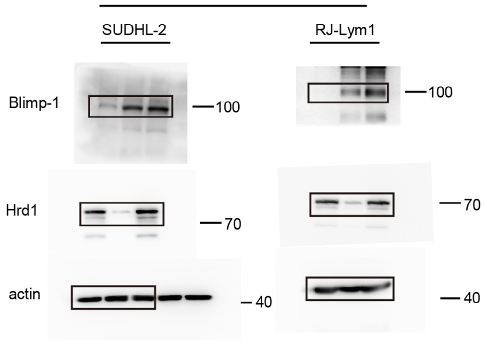

Fig 6d

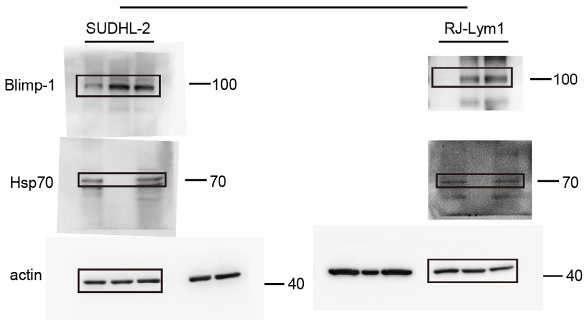

Fig 6e

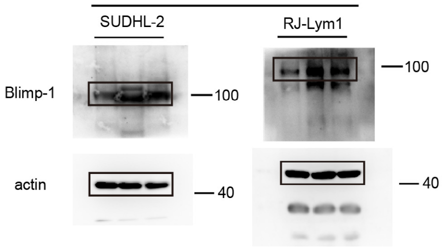

Supplementary Fig. 1d

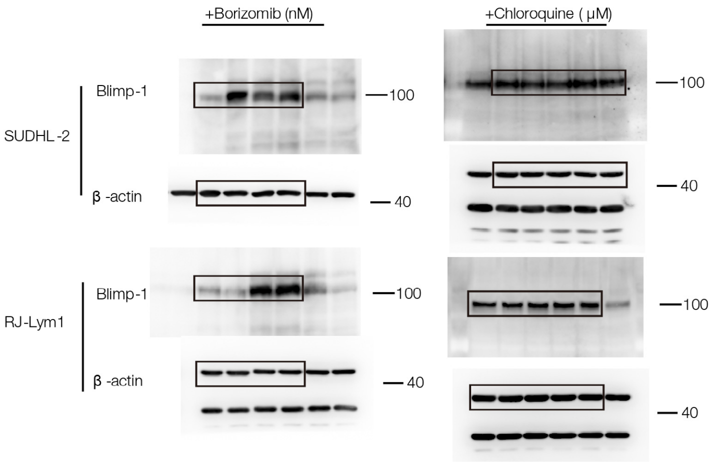

Supplementary Fig.1e

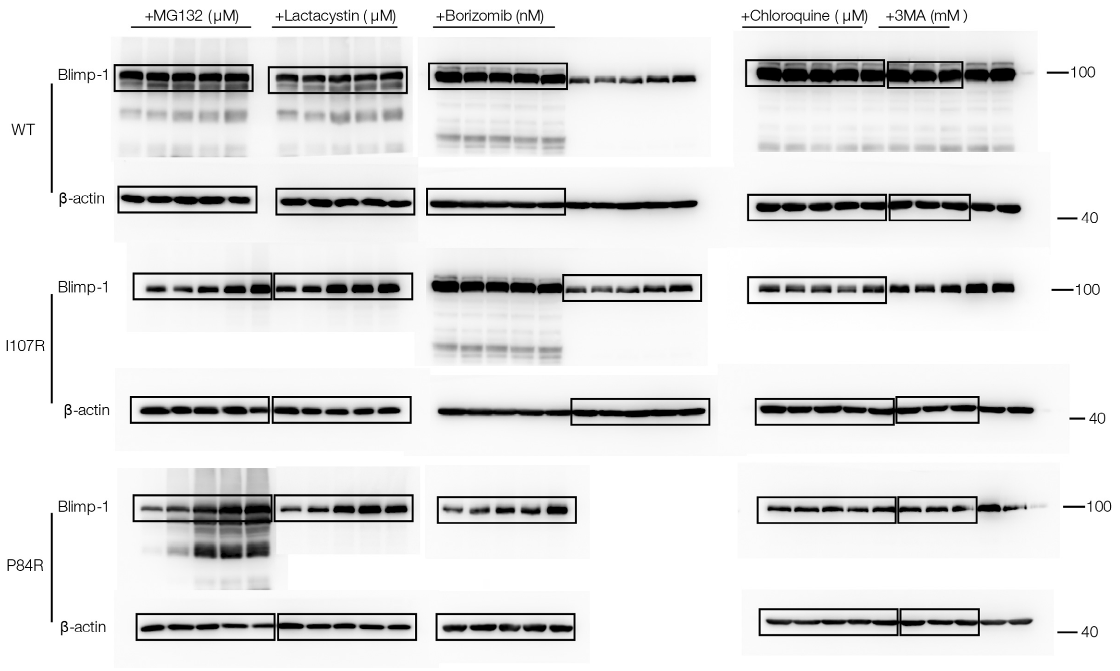

Supplementary Fig. 1f

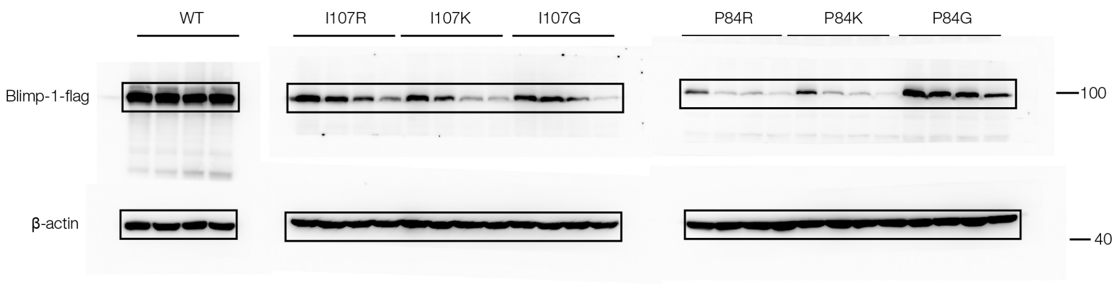

Supplementary Fig. 1g

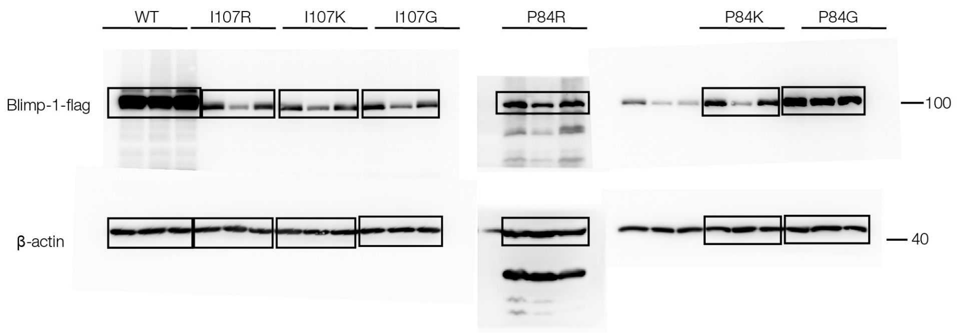

Supplementary Fig. 2a

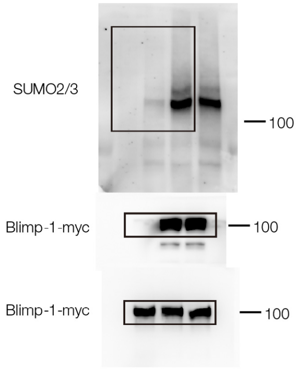

Supplementary Fig. 2b

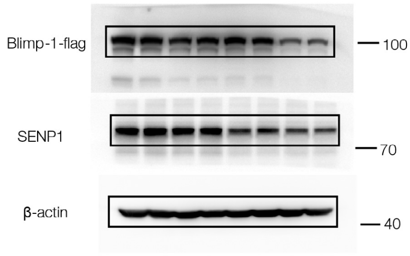

Supplementary Fig. 2d

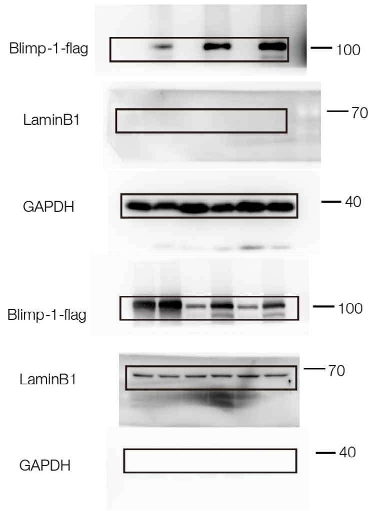

Supplementary Fig. 2h

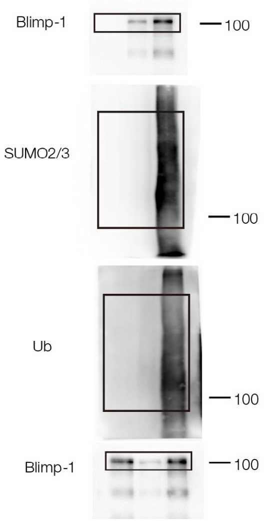

Supplementary Fig. 2i

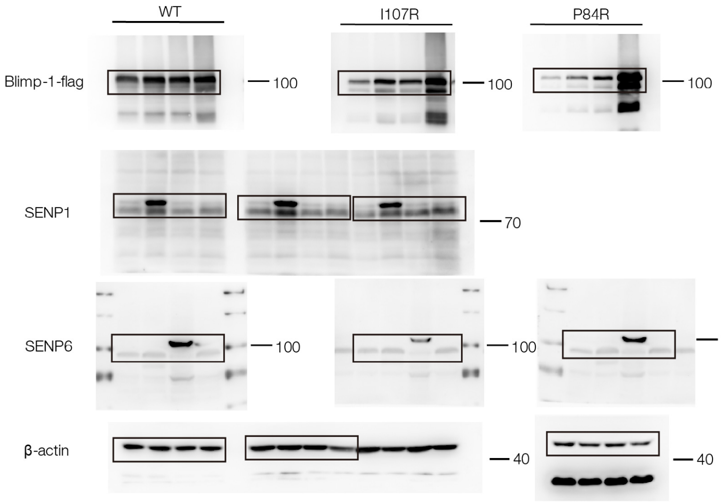

Supplementary Fig. 3a

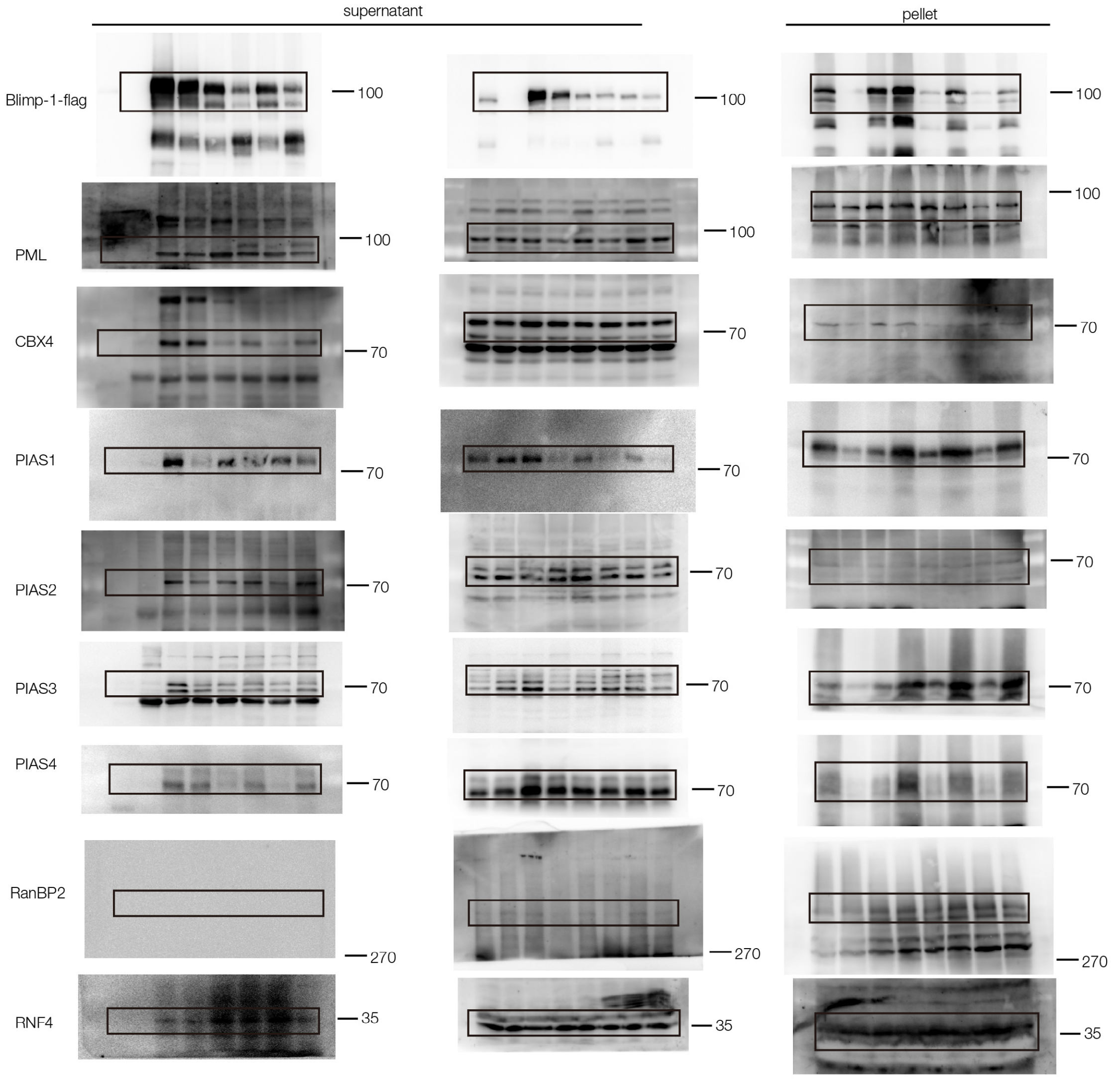

Supplementary Fig. 3c

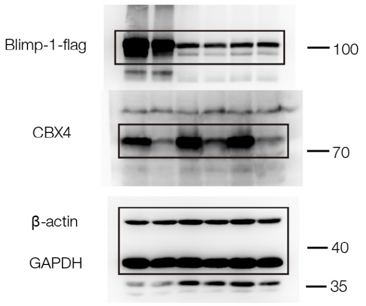

Supplementary Fig. 5a

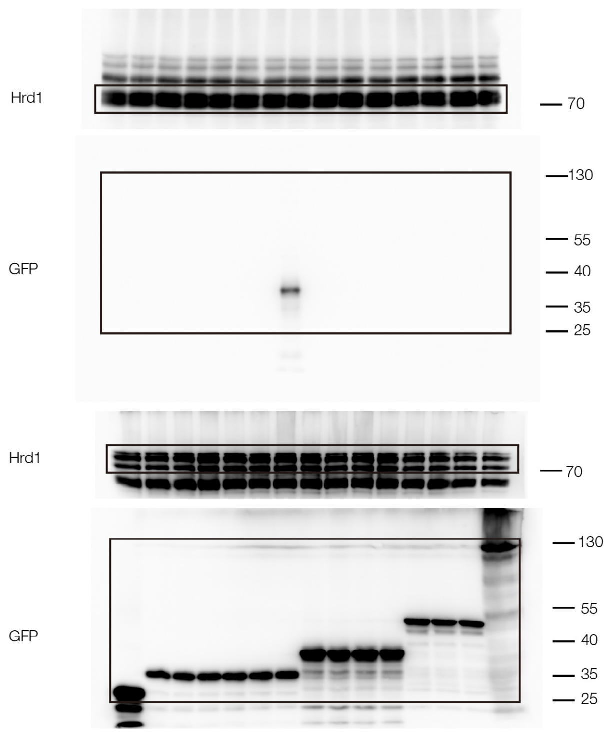

Supplementary Fig. 5e

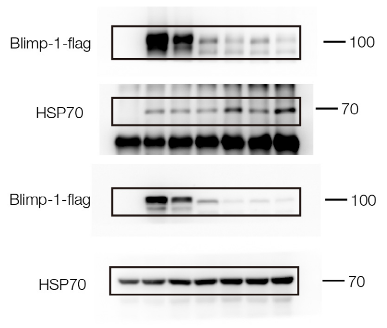

Supplementary Fig. 5d

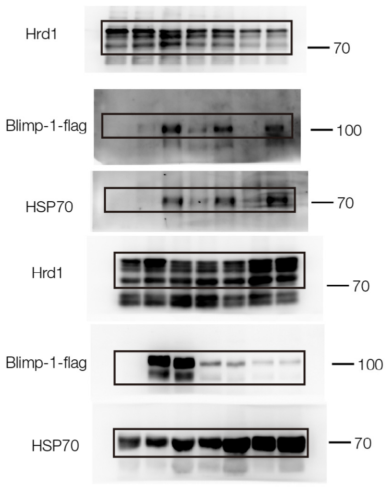

Supplementary Fig. 5f

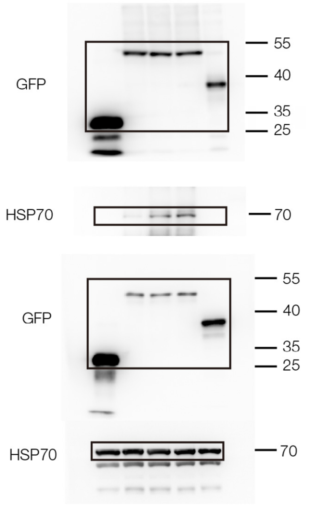

Supplementary Fig. 5i

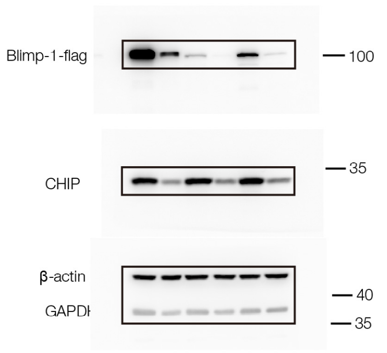

Supplementary Fig. 6a

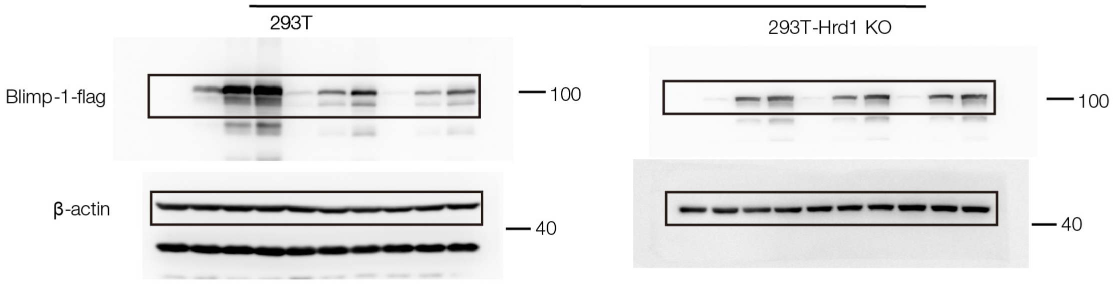

Supplementary Fig. 6b

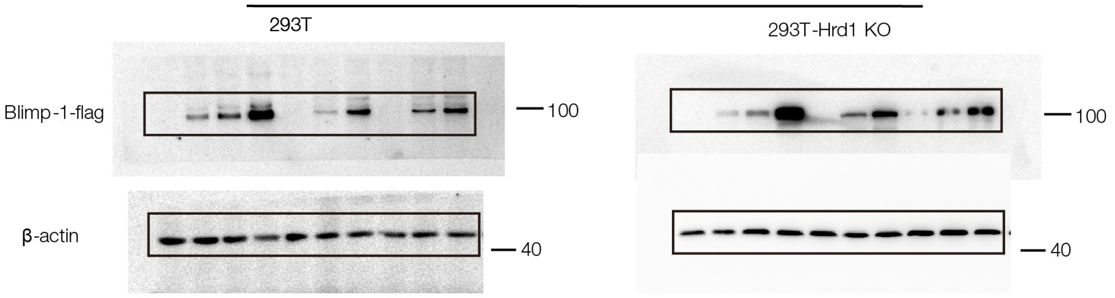

Supplementary Fig. 6f

Supplementary Fig. 6c

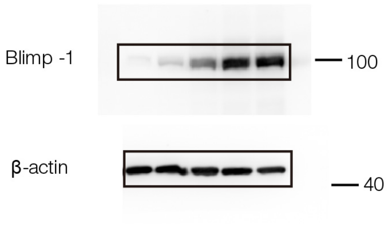

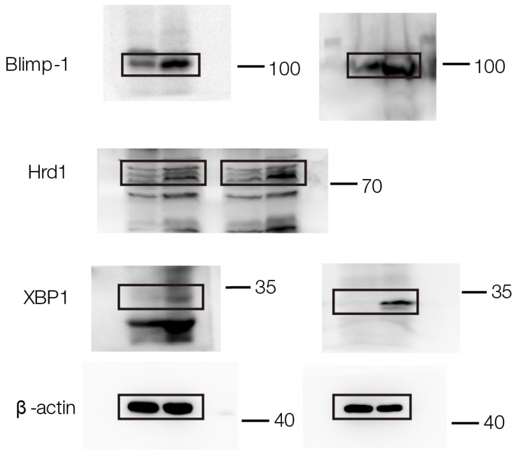

Supplementary Figure 8. The uncropped scans of blots

Supplementary Table 1.

Identification of the proteins physically associating with WT or mutant Blimp-1 during degradation

Protein ID	Gene	Protein	Fold change / P value	
			WT + MG132 VS WT	I107R + MG132 VS I107R
P28066	*PSA5*	HUMAN Proteasome subunit alpha type-5	1.02	2.09 (P < 0.01)
O14818	*PSA7*	HUMAN Proteasome subunit alpha type-7	1.03	1.88 (P< 0.01)
G3V5Z7	*PSMA6*	HUMAN Proteasome subunit alpha type	0.92	1.76 (P< 0.05)
Q59EJ3	*HSPA1A*	HUMAN Heat shock 70kDa protein 1A variant	1.27	1.61 (P< 0.01)

Flag-tagged WT or mutant Blimp-1 proteins were overexpressed in 293T cells, which were treated with MG132 (20 μM) or left untreated for 24 h. The extracts were co-immunoprecipitated with an anti-flag antibody. The co-immunoprecipitated lysates were then analyzed by LC-MS/MS. A fold change > 1.5 was considered a positive association.

Supplementary Table 2.

The estimated hydrophobic surface area of the aa 38-130 fragment or the aa 131-223 fragment in WT and mutant Blimp-1 proteins.

Polypeptide (aa38-223)	Hydrophobic surface (Å) (aa38-130)	Hydrophobic surface (Å) (aa131-223)
**WT**	1507.9±40.3	1136±61
**P84R**	1517.0±50.7	1129.2±51.4
**I107R**	1516.3±51.5	1104.6±72.9

Supplementary Table 3.

The frequencies of N-terminal misfolding mutations that destabilize Blimp-1 via the HSP70-Hrd1 axis in primary ABC/non-GCB DLBCL samples.

Number of ABC/non-GCB DLBCL (primary biosies) (#refernce)	Cases with Blimp-1 point mutations in 1-130aa	Protein instability	Nuclear accumulation restored specifically by VER155008	HSP70 recognition	Restoration of transcriptional regulatory activity in Hrd1 deficiency
67 (#38)	1	1 (P84T)	1 (P84T)	1 (P84T)	1 (P84T)
48 (#28 and #29)	1	1 (I107K)	1 (I107K)	1 (I107K)	1 (I107K)
250 (#19)	13	6 (T23I, A81T, P84L*2, P84S, I117M)	4 (T23I, P84L*2, 84S)	4 (T23I, P84L*2, 84S)	4 (T23I, P84L*2, P84S)

Supplementary Table 4.

The sequences of primer pairs used in Real-Time PCR

Gene	Forward primer	Reverse primer
Blimp-1	GCACACGTTTTGGACCCCTAA	AATGAAGTGGTGAAGCTCCCCT
PAX5	AAGCGCAAGAGAGACGAAGGT	AAGCGCAAGAGAGACGAAGGT
CIITA	GCTGAAGTCCTTGGAAACCCT	CCACGTCGCAGATGCAGTTAT
ID3	GAAATCCTACAGCGCGTCATC	CAGTGGCAAAAGCTCCTTTTG
c-Myc	TCCCTCCACTCGGAAGGACTAT	CAAGACGTTGTGTGTTGGCCT
IgJ	GGAGTCCTGGCGGTTTTTATT	TCGGAAGAACGGATGATCCT
18S	CGCGGTTCTATTTTGTTGGTTT	TTCGCTCTGGTCCGTCTTG
